# T Cell-Specific Inactivation of the PI3K p110α Catalytic Subunit: Effect in T Cell Differentiation and Antigen-Specific Responses

**DOI:** 10.3390/ijms26020595

**Published:** 2025-01-12

**Authors:** Alejandro C. Briones, Laura del Estal, Cristina Villa-Gómez, Verónica Bermejo, Isabel Cervera, Pedro Gutiérrez-Huerta, María Montes-Casado, Sagrario Ortega, Mariano Barbacid, José María Rojo, Pilar Portolés

**Affiliations:** 1 Centro Nacional de Microbiología, Instituto de Salud Carlos III (ISCIII), Majadahonda, 28220 Madrid, Spain; 2 Department of Immunology, Complutense University School of Medicine, i+12 Research Institute, 28040 Madrid, Spain; 3 Centro Nacional de Investigaciones Oncológicas (CNIO), 28029 Madrid, Spain; 4 Centro de Investigaciones Biológicas Margarita Salas, Consejo Superior de Investigaciones Científicas (CSIC), 28040 Madrid, Spain

**Keywords:** class I phosphoinositide 3-kinases, PI3 kinases, T lymphocyte, thymus, Treg, Tfh, antigen response

## Abstract

Class IA PI3K p110δ and p110α subunits participate in TCR and costimulatory receptor signals involved in T cell-mediated immunity, but the role of p110α is not completely understood. Here, we analyzed a mouse model of the Cre-dependent functional inactivation of p110α (kinase dead) in T lymphocytes (p110αKD-T, KD). KD mice showed increased cellularity in thymus and spleen and altered T cell differentiation with increased number of CD4^+^CD8^+^ DP thymocytes, enhanced proportion of CD4^+^ SP lymphocytes linked to altered apoptosis, lower Treg cells, and increased AKT and ERK phosphorylation in activated thymocytes. In the spleen, the percentages of CD4^+^ Treg cells and CD8^+^ naive lymphocytes were reduced. In vitro, the differentiation of CD4^+^ cells from p110αKD-T mice showed lower induced Treg (iTreg) cell yield or IL-10 secretion. Moreover, Tfh cell yield, IL-21 secretion, and PI3-K-dependent elongation were hampered, as was Erk and Akt activation. Th1 or Th17 differentiation in vitro was not altered. The immunization of p110α-KD-T mice with KLH protein antigen induced an enhanced proportion of CXCR5^+^ CD4^+^ cells and germinal center B cells, increased ICOS expression in CD4^+^ cells, or IFN-γ secretion upon antigen re-activation in vitro. However, anti-KLH antibody responses in serum was similar in WT or p110α KD mice. These data show that T cell-specific p110α inactivation alters T cell differentiation and function.

## 1. Introduction

Signals mediated by class I phosphoinositide 3-kinases (PI3Ks) are critical to the control of many cellular processes relevant to immune function including metabolism, proliferation, and differentiation and play a major role in inflammation and carcinogenesis (reviewed in [1,2,3,4,5,6,7,8,9]). Class I PI3Ks phosphorylate the 3-OH group of the inositol ring in phosphoinositides (PI(4,5)P2) with the inositol phosphorylated in the 4-OH and 5-OH groups producing PI(3,4,5)P3 (PIP3)). Class I PI3Ks are typically activated when heterodimers of regulatory and catalytic subunits in the cytoplasm are recruited close to cell membranes. This is achieved by the binding of regulatory subunits to sequence motifs containing phosphorylated tyrosine (in class IA PI3K) or G-protein receptor subunits (in class IB PI3K). Class IA PI3K catalytic subunits in mammals are p110α, p110β, or p110δ and can form dimers with distinct regulatory subunits (p85α, p50α, p55α, p85β, and p55γ). The abundance of regulatory and catalytic subunits differs among cells and tissues, dictating the importance to their functions and the possibility of selective modulation through inhibition or deletion. Relevant to this study, whereas the class IA catalytic subunits p110α and p110β are widely expressed, class IA p110δ subunits (and class IB p110γ catalytic subunits) are mainly expressed by cells of hematopoietic origin [1,8,10].

Signals mediated by class I PI3Ks are activated in T and B lymphocytes by many membrane receptors including antigen receptors and costimulatory molecules. Particularly, the activation of PI3K in T cells mediated by receptors of the CD28 family (like CD28 and ICOS) is essential to the development of normal and pathogenic immune responses. In line with its pattern of expression in leukocytes, the catalytic subunit p110δ is needed for appropriate T cell differentiation and function in vitro and in vivo, as shown by isoform-specific inhibition, deficiency, or inactivation [11,12,13,14,15,16,17,18,19,20,21,22,23,24,25].

Since T lymphocytes express similar levels of p110α and p110δ class IA PI3Ks, and p110α binding to PI3K regulatory subunits is stronger than that of p110δ [26], the role of p110α in T cell functions needs to be examined. However, this is hampered by the important role of p110α PI3K in angiogenesis, which produces embryonic lethality in knock-out animal models. Thus, it is necessary to conditionally modify p110α PI3K expression to determine its function in T cells.

Our previous data using CD4^+^ and CD8^+^ T lymphocytes deficient in p110α showed enhanced responses as well as Th1 and Th17 differentiation in vitro. Furthermore, p110α-deficient Tfh cells differentiated in vitro showed enhanced cytokine secretion but impaired motility, and Treg differentiation was inhibited [17]. In vivo, mice with p110α-deficient T cells show enhanced cytokine secretion, Tfh differentiation, and immune responses to protein antigens or tumors, as well as altered homeostasis of some CD4^+^ T populations [17,27,28].

Intriguingly, the proportion of Tregs in mice with p110α-deficient T cells changes over time, leading to enhanced or suppressed responses depending on the illness model considered and the age of the mice [17,27,28]. Results with Treg-specific loss of p110α confirm its relevance to Treg development and function [25]. Thus, the overall effect of T-cell-specific deletion of p110α can be beneficial or detrimental depending on the specific response and parameter considered.

These results indicate the convenience of exploring p110α-specific inhibitors, alone or in combination, in anti-inflammatory interventions. Moreover, the impact on the immune responses of p110α-specific inhibitors used in anticancer therapies needs to be assessed. We have previously observed that the activation, differentiation, and motility of T lymphocytes in vitro can be inhibited to different degrees by p110α blockers. This fosters the effect of p110δ and DNA protein kinase inhibitors, with wide differences in sensitivity to inhibition depending on the nature of the cells (i.e., naïve, blasts) and the cytokine considered [16,26,29]. For instance, IFN-γ, but not IL-10 secretion, is very sensitive to inhibition by PI3K inhibitors, and IL-21 secretion from Tfh cells is efficiently blocked only by combining the inhibition of p110α and p110δ. The administration of these inhibitors in vivo inhibits immune responses against protein antigens. Inhibitors also attenuate symptoms in pre-clinical models of autoimmune diseases like collagen-induced arthritis or experimental autoimmune encephalitis (EAE) [16,26,29].

To more precisely analyze the role of p110α in T lymphocyte development and function, in this paper, we develop a new mouse model of the Cre-dependent, T lymphocyte-specific inactivation of p110α (kinase dead; p110αKD-T). The functional inactivation of p110α causes altered cellularity and cell signaling in the thymus and spleen. Altered differentiation and function of some mature T cell subsets including Tfh and Treg can also be observed in vitro and in immune responses to protein antigens in vivo.

## 2. Results

### 2.1. Altered Thymus Differentiation in p110αKD-T Mice

**Figure 1:**
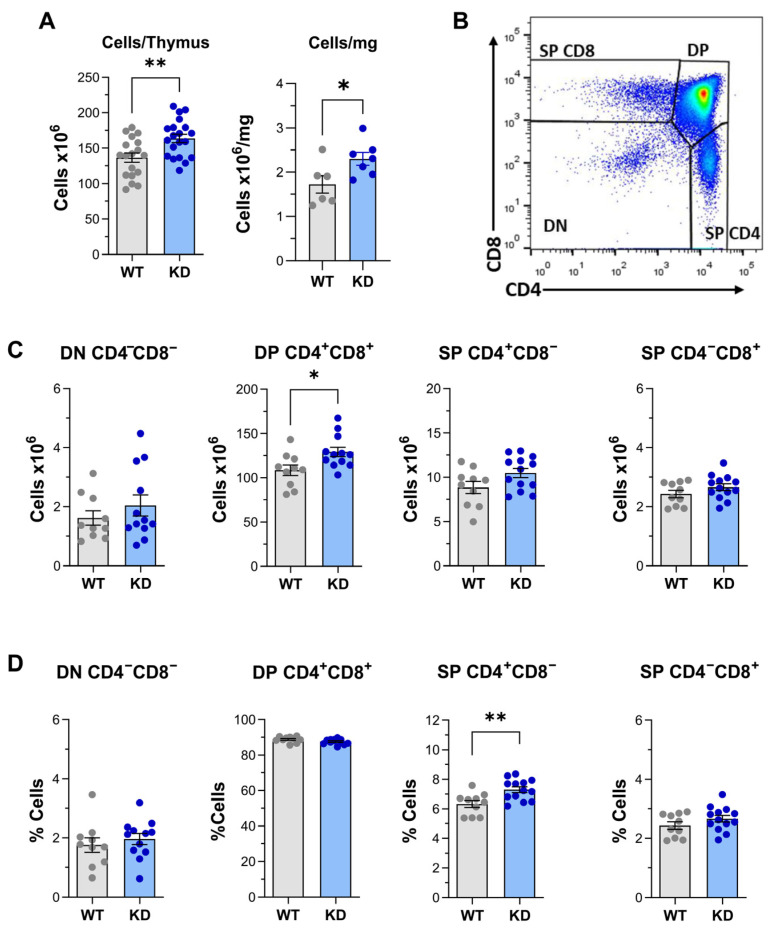
Thymus: total cells and main subpopulations. Number of thymus cells and thymus subpopulations as determined by flow cytometry of thymocytes from wild-type (WT) mice, or from mice with T cells expressing an inactive, kinase-dead form of PI3K p110α (p110αKD-T, KD). (**A**) Total cells per thymus (left) or per mg of tissue (right) in WT (grey bars) or KD (blue bars) mice. (**B**) Analysis of thymus maturation stages as determined by flow cytometry (density dot plot) using CD4 and CD8 staining. DN: CD4^−^CD8^−^ thymocytes; DP: CD4^+^CD8^+^ thymocytes; SP CD4: CD4^+^CD8^−^ thymocytes; SP CD8: CD4^−^CD8^+^ thymocytes. Number of cells (**C**) and percentage of thymocytes (**D**) within the DN, DP, SP CD4, or SP CD8 subpopulations in the thymus from WT (grey) or KD (blue) mice. Individual mice values as well as the mean ± SEM for each group are depicted. Only significant differences between groups are indicated, as determined by the Student’s *t* test (* *p* < 0.05, ** *p* < 0.01). (**A**) Left, WT *n* = 18, KD *n* = 20; right, WT *n* = 6, KD *n* = 7. (**C**,**D**) WT *n* = 10, KD *n* = 12.

The development of T lymphocytes requires a process of differentiation, selection, and maturation in their primary organ, the thymus. When compared with littermates expressing the wild-type protein (WT), mice expressing inactive p110α PI3K in the T cell lineage (p110αKD-T) did not show clear anatomic changes in primary or secondary lymphoid organs. However, when thymuses were compared, the number of cells per thymus or referred to tissue weight was significantly higher in p110αKD-T (Figure 1A).

To determine the thymic populations contributing to this phenomenon, thymus T cell differentiation stages were further analyzed by flow cytometry (Figure 1B–D, see also Figure S1A for gating strategy). The development of conventional T cells expressing αβTCR proceeds through three main stages in which thymocytes may be broadly characterized, based on their surface expression of the co-receptors CD4 and CD8. The most immature population is known as double negative (DN, CD4^−^CD8^−^) cells, which progressively give rise to an intermediate double positive (DP, CD4^+^CD8^+^) phase and eventually, to a mature phase of single positive (SP) CD4^+^ or CD8^+^ cells. These broad stages reach further resolution based on the expression of additional cell surface markers, as CD44 and CD25 in DN thymocytes, CD3/CD5 in DP thymocytes, and CD3/CD24 in SP T cells (Reviewed in [30,31,32]) (see Figure S1).

As shown in Figure 1C,D, the number or the frequency of CD4^−^CD8^−^ DN subpopulations did not show significant alterations, confirming the CD4 linkage of p110α mutation in this model. When checked for cell numbers, the number of CD4^+^CD8^+^ DP cells per thymus was significantly enhanced in p110αKD-T mice; this indicated that that population was the main contributor to the higher cellularity observed in the p110αKD-T thymus (Figure 1C). Furthermore, the frequency of CD4^+^ SP cells was enhanced in these mice, but CD8^+^ SP cells were not significantly altered in the p110αKD-T thymus (Figure 1C,D).

Thymocytes suffer various processes of selection (see Figure S1B), the first of them, at the DN3 stage, is named β-selection as only DN3 cells with functionally recombined TCRβ develop further to express a pre-TCR (composed of TCRβ, pre-TCRα, and CD3 subunits). The pre-TCR emits survival signals which rescue cells from apoptosis reaching the DN4 stage. The pre-TCR triggers rapid cell proliferation and progression to an intermediate CD4^−^CD8^+^TCR^−^ ISP stage and subsequently into CD4^+^CD8^+^ DP blasts (reviewed in [30]). At the DP stage, thymocytes rearrange the TCRα locus to express a mature clonotypic TCRαβ antigen receptor and may recognize self-peptide-MHC (p-MHC) ligand. The high affinity recognition of p-MHC triggers cell death by apoptosis through another selection process, the negative selection. Thymocytes with very low self-reactivity are also eliminated, due to insufficient survival signals (death by neglect). Moderate affinity recognition triggers a third type of selection process, the positive selection, accompanied by the increased expression of CD5, CD69, and the TCR. Cells positively selected (approximately 5%) pass through a transitional stage of high expression of CD24 to go into the mature SP compartment (SP-CD4^+^ or SP-CD8^+^), with progressively lower expression of CD24 (reviewed in [30,31,32]).

**Figure 2:**
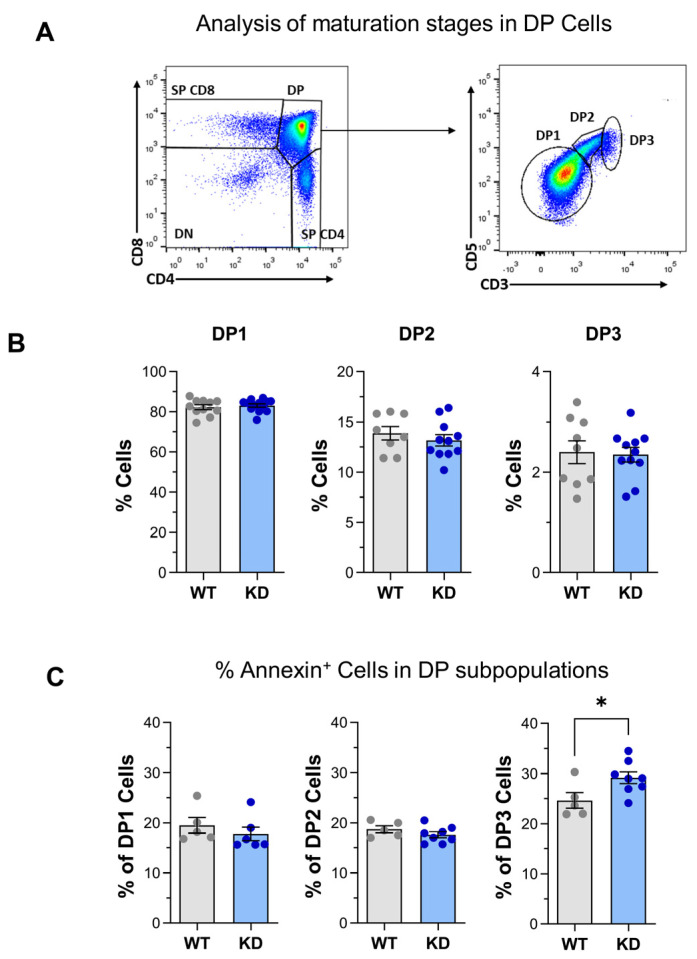
Maturation of CD4^+^CD8^+^ DP thymocytes during thymic differentiation. (**A**) Strategy for the analysis of thymus maturation stages as determined by flow cytometry (density dot plots). DP1–DP3 indicate successive maturation stages of CD4^+^CD8^+^ DP thymocytes, as determined by the expression levels of CD3 and CD5. (**B**) Percentage of DP1, DP2, and DP3 CD4^+^CD8^+^ cells in thymus of WT (grey bars) or KD (blue bars) mice. (**C**) Percentage of apoptotic cells within DP1, DP2, and DP3 CD4^+^CD8^+^ in WT (grey bars) or KD (blue bars) thymocytes, as determined by staining with Annexin V. Data from individual WT (grey dots) or p110αKD-T mice (KD, blue dots) as well as the mean ± SEM for each group are depicted. Significant differences between groups are shown, as determined by the Student’s *t* test (* *p* < 0.05). (**B**) WT *n* = 9, KD *n* = 11. (**C**) WT *n* = 5, KD *n* = 8.

To further analyze the development of T lymphocytes in our p110αKD-T model, the successive maturation stages within CD4^+^CD8^+^ DP thymocytes (DP1-DP3) were analyzed based on the expression of CD3 and CD5 [33,34,35,36] (Figure 2A,B). DP1 thymocytes (CD5^low^CD3^low^) are precursors of CD5^high^CD3^int^ DP2. These differentiate to CD4^+^ SP T cells or proceed to the DP3 (CD5^int/high^CD3^high^) stage where they differentiate into CD8^+^ T cells [36].

No changes were observed in the percentage of DP1–DP3 subpopulations (Figure 2B) indicating that the increased cellularity in this compartment is common to the three maturation stages. Interestingly, apoptosis was enhanced in the more mature DP3 cells from p110αKD-T mice, as shown by Annexin V staining (Figure 2C).

**Figure 3:**
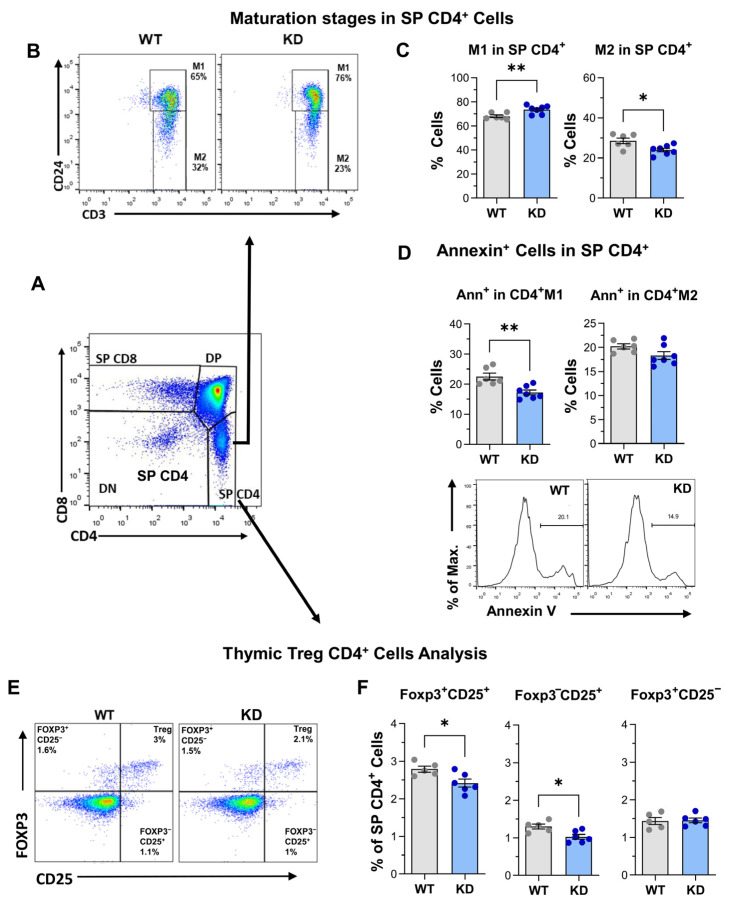
Maturation stages of SP CD4^+^ thymocytes and thymus Treg in WT and p110αKD-T SP CD4^+^ thymocytes. CD4^+^CD8^−^ SP CD4^+^ thymocytes (**A**, density dot plots) from WT (grey) or p110αKD-T mice (KD, blue) were analyzed for CD3 and CD24 expression (**B**, density dot plots) to determine the percentage of immature SP thymocytes (M1, **C** (left)) and mature M2 cells (**C** (right)). (**D**) Annexin V (Ann) staining was used to assess the percentage of apoptotic cells within M1 (left) and M2 (right) CD4^+^ SP thymocytes. Representative cytometry histograms are shown. (**E**,**F**) Staining with anti-CD25 and anti-Foxp3 was used to determine the percentage of Foxp3^+^CD25^+^ (Treg cells) and Foxp3^−^CD25^+^ and Foxp3^+^CD25^−^ pre-Treg cells. Representative density dot plots are shown. Data from individual WT (grey dots) or p110αKD-T mice (KD, blue dots) and the mean ± SEM for each group are shown. Significant differences are shown, as determined by the Student’s *t* test (* *p* < 0.05, ** *p* < 0.01). (**C**,**D**) WT *n* = 6, KD *n* = 7; (**F**) WT *n* = 5, KD *n* = 6.

The maturation of CD4^+^ SP thymocytes was then tracked based on CD24 expression levels [36,37] (see Figure S1), i.e., CD24^high^ immature CD4^+^ SP thymocytes (M1) or CD24^low^ mature CD4^+^ SP cells (M2) (Figure 3A,B).

Mutant mice showed an enhanced proportion of immature CD4^+^ SP M1 cells but a lower proportion of the more mature M2 population (Figure 3C, top panel). This is in line with lower apoptotic cells among p110αKD-T M1 cells (Figure 3D, lower panels).

A similar analysis of CD8^+^ SP thymocytes showed no differences in the percentages of immature or mature subpopulations, apoptotic cells (Figure S2). The percentage and characteristics of the small population of immature CD3^−^CD8^+^ SP cells (iSP8) that are precursors of CD4^+^CD8^+^ DP thymocytes were also unaltered (Figure S2).

As expected, no changes were observed in the CD4^−^CD8^−^ DN differentiation stages (DN1-DN4), as determined by CD44 and CD25 expression (Figure S3A,B).

### 2.2. Treg Differentiation in the Thymus

Possible differences in the subpopulation of Treg cells within CD4^+^ SP thymocytes were also determined. As shown in Figure 3E,F, the percentage of Foxp3^+^CD25^+^ Treg cells was significantly lower in p110αKD-T mice; this was also observed in the Foxp3^−^CD25^+^ cells that are considered one possible precursor population for Treg cells in the thymus. As we see below, altered Treg differentiation was consistently observed in distinct conditions in p110αKD-T mice. The analysis of apoptosis in Treg FoxP3^+^CD25^+^ showed no significant differences between p110αKD-T mice and their WT counterparts (Figure S3C); however, when proliferation was analyzed by intracellular Ki67 expression, lower levels of this marker were found in p110αKD thymic Treg (Figure S3C).

### 2.3. Early Activation Signals in Thymocytes

**Figure 4:**
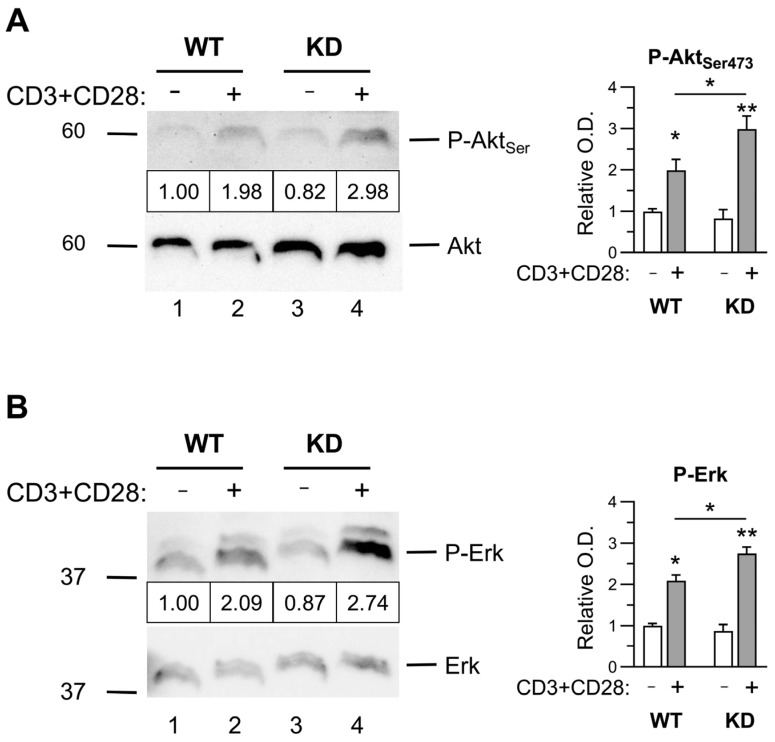
Intracell signaling in thymocytes. Comparison of Akt and MAP kinase phosphorylation in activated WT and p110αKD-T thymocytes (KD). Cells were activated for 10 min with anti-CD3 plus anti-CD28 antibodies, as indicated. Immunoblot of cell lysates was performed with antibodies specific for (**A**) Phospho-Akt_Ser473_ or (**B**) Phospho-Erk1/2_Thr202/Tyr204_. Graphs indicate relative O.D considering the signal of mock-activated WT thymocytes as 1, using Akt (**A**) or Erk2 (**B**) as internal loading controls. Asterisks on columns indicate significant differences of activated versus control cells; asterisks on lines show significant differences between WT and p110αKD-T cells in the same conditions, as determined by an ANOVA (* *p* < 0.05, ** *p* < 0.01).

Since the data of p110αKD-T thymuses suggest an enhanced generation of DP cells as well as an altered positive selection of CD4^+^ SP and thymus Treg differentiation, we analyzed TCR- and PI3K-dependent signals that might be responsible for these effects. Our results indicated higher Akt and Erk activation in p110αKD-T thymocytes activated with anti-CD3 plus anti-CD28 antibodies, as shown by their increased phosphorylation (Figure 4A,B).

These data are in agreement with the role of Erk in the positive selection of DP thymocytes and lineage commitment of CD4^+^ SP [38,39], and thymocyte survival could be favored by enhanced Akt activation [40,41] that might block thymus Treg cell differentiation [42].

No significant differences were observed in mTORC1 phosphorylation, and P38 phosphorylation was barely enhanced by CD3/CD28 activation in thymus cells (Figure S4A,B). It should be noted that WT and p110αKD-T thymocytes had no significant differences in the expression levels of the p110α or p110δ catalytic class IA PI3K subunits (Figure S4C). Like thymus cells, mature CD4^+^ spleen T cells from WT or p110αKD mice expressed comparable levels of p110α or p110δ subunits (Figure S4D).

### 2.4. Impact of p110αKD Expression in Treg and Other Spleen Cell Populations

**Figure 5:**
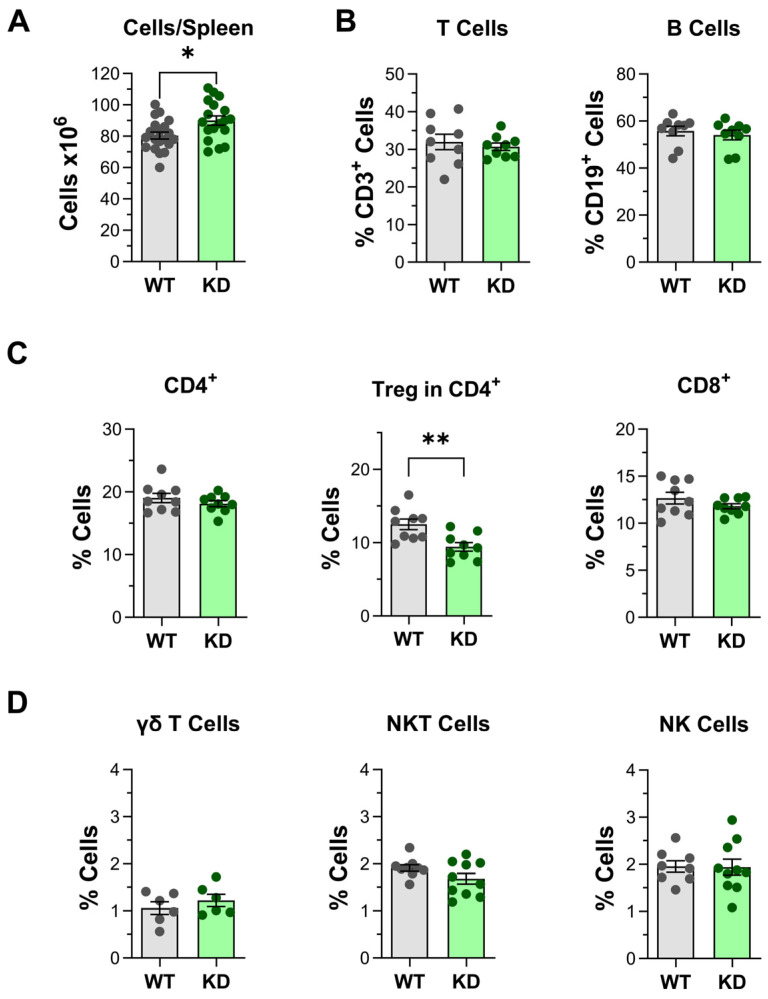
Analysis of total cells and T, B, and NK cells and subpopulations in the spleen. Number of spleen cells (**A**) and percentage of T (CD3^+^) and B (CD19^+^) lymphocytes (**B**), T cell subpopulations (CD4^+^, CD25^+^Foxp3^+^ Treg, CD8^+^, γδ^+^ CD3^+^, and NK1.1^+^CD3^+^ (NKT) and NK lymphocytes (NK1.1^+^CD3^−^) (**C**,**D**) in the spleen of wild-type (WT, grey) or p110αKD-T (KD, green) mice, as determined by flow cytometry. Data from individual WT (grey dots) or p110αKD-T mice (KD, green dots) and the mean ± SEM for each group are shown. Significant differences between WT and KD mice are shown, as determined by the Student’s *t* test (* *p* < 0.05, ** *p* < 0.01). (**A**) WT *n* = 20, KD *n* = 18; (**B**–**D**) WT *n* = 9, KD *n* = 9, except γδ T cells (WT *n* = 6, KD *n* = 6).

Considering the quantitative and qualitative changes observed in thymus development, the effects of p110α inactivation in spleen cell populations were analyzed. Like the thymus, spleens from p110αKD-T mice had a small but significant increase in cell numbers (Figure 5A).

No significant alterations were observed in total T or B lymphocytes (Figure 5B), or in the percentage of CD4^+^, CD8^+^, γδ T cells, NKT, or NK lymphocytes (Figure 5C,D).

Interestingly, as observed in the thymus, the percentage of Foxp3^+^CD25^+^ Treg among CD4^+^ T cells was significantly lower in p110αKD-T spleens (Figure 5C). In contrast, in the CD4^+^ T cell population the percentages of naïve and memory lymphocytes were not altered in p110αKD-T spleens (Figure S5). The percentage of naïve (CD62L^+^CD44^low^) CD8^+^ T cells was slightly yet significantly lower in the spleens from mutant mice (Figure S5). As noted above, the expression of p110α or p110δ catalytic subunits was not significantly different in lysates of CD4^+^ T spleen cells from WT and p110αKD-T mice (Figure S4D).

### 2.5. CD4^+^ Treg Differentiation “In Vitro”

**Figure 6:**
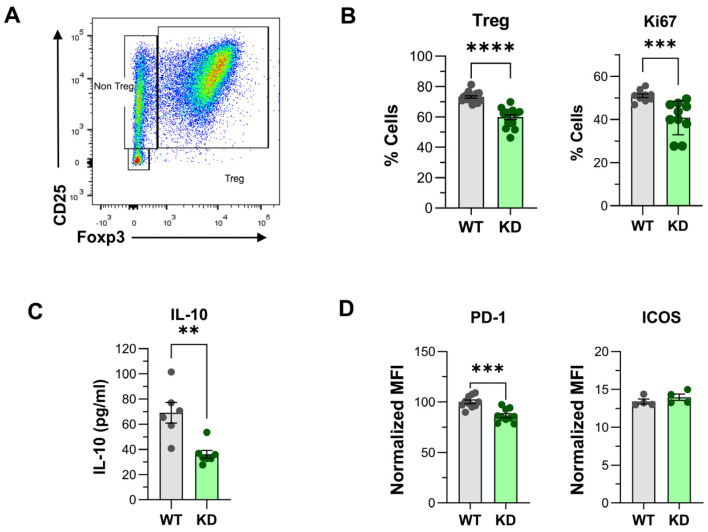
In vitro Treg differentiation of WT and p110αKD-T CD4^+^ T lymphocytes. (**A**) Representative density dot plot of naïve CD4^+^ T lymphocytes after 72 h of culture activated for Treg differentiation. Treg differentiation was assessed by CD25^+^Foxp3^+^ expression within CD4^+^ T cells. (**B**) Percentage of CD25^+^Foxp3^+^ (left panel) and cells positive for the proliferation marker Ki67 (right panel) in Treg cultures from WT (grey) or p110αKD-T (KD, green) mice. (**C**) IL-10 in the supernatants and (**D**) expression of PD-1 and ICOS in cells of Treg cultures from WT and KD mice. Data from individual WT (grey dots) or p110αKD-T mice (green dots) and the mean ± SEM for each group are shown. Median of fluorescence intensity (MFI) was normalized against MIF in isotype control staining. (**B**) Left: WT *n* = 16, KD *n* = 16; right: WT *n* = 10, KD *n* = 10; (**C**) WT *n* = 6, KD *n* = 7; (**D**) Left: WT *n* = 10, KD *n* = 10; right: WT *n* = 4, KD *n* = 4. Significant differences between WT and KD mice were determined by the Student’s *t* test (** *p* < 0.01, *** *p* < 0.001, **** *p* < 0.0001).

Since the percentage of CD4^+^ Tregs was lower in the thymus and the spleen from p110αKD-T mice, possible alteration of Treg differentiation in vitro was assessed. Indeed, the Treg differentiation of p110αKD-T CD4^+^ T cells was altered as indicated by a lower percentage of CD25^+^Foxp3^+^ Treg cells in the culture, cell proliferation (measured as Ki67 expression), or PD-1 expression and IL-10 secretion (Figure 6A–D).

Expression level of other relevant functional markers, like ICOS, was similar in WT or p110αKD-T iTregs (Figure 6D).

### 2.6. Effect of p110α Inactivation on CD4^+^ Th1, Th17 and Tfh Cell Differentiation In Vitro

The functional impact of p110α inactivation in T lymphocytes was also studied by comparing cells from WT or p110αKD-T mice cultured in vitro under conditions favoring the differentiation of distinct T helper subsets. We did not observe significant differences between WT and p110αKD-T CD4^+^ T cells when naïve cells were cultured under conditions promoting the generation of Th1 or Th17 cells (Figure S6).

**Figure 7:**
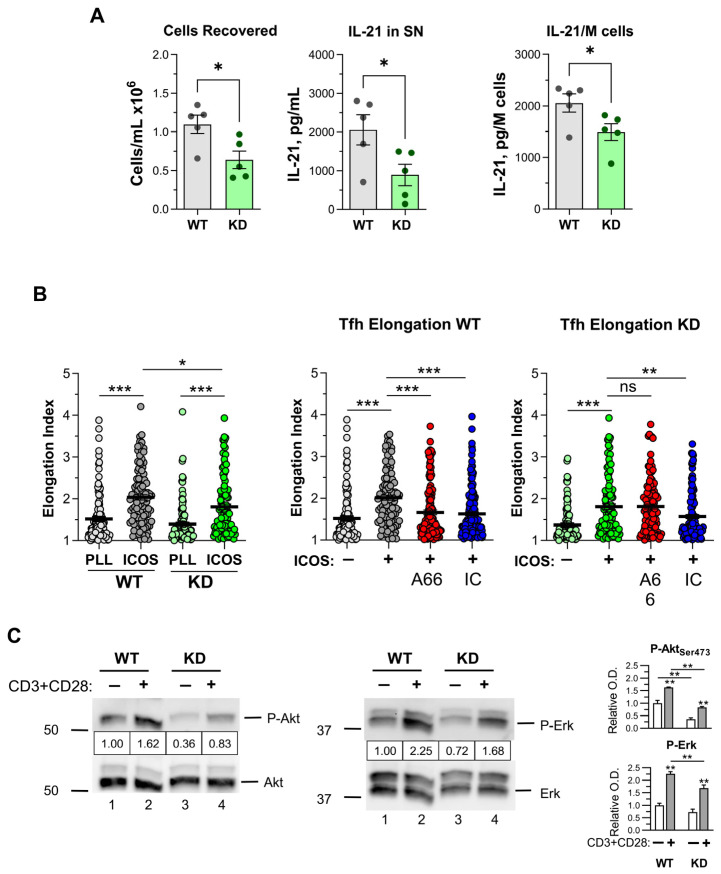
Differentiation of CD4^+^ T cells into T follicular helper (Tfh) cells *in vitro* and T cell signaling. (**A**) Naive CD4^+^ T lymphocytes from WT (grey) or p110αKD-T (KD, green) were differentiated for 72 h in vitro with anti-CD3 plus anti-CD28 antibodies in the presence of IL-6. Cells recovered from the cultures were counted (left panel) and IL-21 content in the supernatants (center panel) and IL-21 per 10^6^ cells in the culture (right panel) were determined. Data from individual WT (grey dots) or p110αKD-T mice (green dots) and the mean ± SEM for each group are also shown (WT *n* = 5, KD *n* = 5). Significant differences between WT and KD mice were determined by the Student’s *t* test (* *p* < 0.05). (**B**) Elongation of WT (grey circles) and KD (green circles) Tfh cells differentiated in vitro induced by Poly-L-Lys (PLL, light symbols) or anti-ICOS (ICOS, dark symbols), as shown in the left panel. The elongation of WT cells (center panel) and KD cells (right panel) in the presence of vehicle (DMSO, grey circles), or inhibitors specific to PI3-kinase p110α (A66, 1 μM, red circles) and p110δ (IC87114, 5 μM, blue circles) was also determined. Mean ± SE of the Elongation Index in >100 individual cells is shown. Significant differences (* *p* < 0.05, ** *p* < 0.01, *** *p* < 0.001) were determined by an ANOVA. (**C**) Akt and MAP kinase phosphorylation in activated WT and p110αKD-CD4^+^ Tfh blasts. Cells were activated 10 min with anti-CD3 plus anti-CD28 antibodies or control antibodies as shown in the figure. Immunoblots of cell lysates were performed with antibodies specific to Phospho-Akt_Ser473_ (left) or Phospho-Erk1/2_Thr202/Tyr204_ (center). Membranes were probed anti-Akt (left) and anti-Erk2 antibodies as loading controls; the relative O.D. in each case is shown considering the signal of mock-activated WT as 1. Asterisks on columns indicate significant differences of activated versus control cells; asterisks on lines show significant differences between WT and p110aKD-T cells in the same conditions, as determined by an ANOVA (** *p* < 0.01).

In contrast, cultures of p110αKD-T CD4^+^ T cells under conditions favoring Tfh generation had a significantly lower number of cells than WT cultures, and the secretion of the Tfh cytokine IL-21 was hampered (Figure 7A).

Since ICOS ligands induce the elongation of Tfh and other ICOS^+^ cells in a PI3K-dependent fashion [17,26], this property was also analyzed. As shown in Figure 7B, the elongation of p110αKD-T CD4^+^ Tfh cells induced by plate-bound anti-ICOS antibodies was significantly lower than the elongation of WT Tfh cells. Furthermore, whereas both p110α-(A66) and p110δ-specific (IC87114) PI3K inhibitors inhibited the elongation of Tfh from WT cells, only a p110δ-specific PI3K inhibitor inhibited p110αKD-T Tfh cell elongation, confirming the specificity of the effect (Figure 7B).

### 2.7. Early Signaling in CD4^+^ T Cells

PI3K activity mediates many signaling pathways in T lymphocytes, including TCR/CD3 activation. To assess the impact of inactivated p110α in mature T CD4^+^ cell signaling, WT and p110αKD-T CD4^+^ Tfh blasts were obtained and compared for the activation of the Akt kinase that is a direct target of class I PI3K activity as well as Erk and P38 MAP kinases. Blasts were activated by anti-CD3 plus anti-CD28 antibodies, and the activation of Erk, P38, and Akt was determined by immunoblot of cell lysates with phospho-specific antibodies (Figure 7C). In WT cells, there was enhanced phosphorylation of Erk and Akt upon activation, which was lower in activated p110αKD-T cells. These data are in contrast with the enhanced early signals observed in thymocytes but fit with our previous data on the effect of p110α inhibition in T cell lines [26]. On the other hand, P38 and mTORC1 phosphorylation were not significantly altered in resting or activated WT or p110αKD-T cells (Figure S7).

### 2.8. Immune Response Against KLH Protein in p110αKD-T Mice

**Figure 8:**
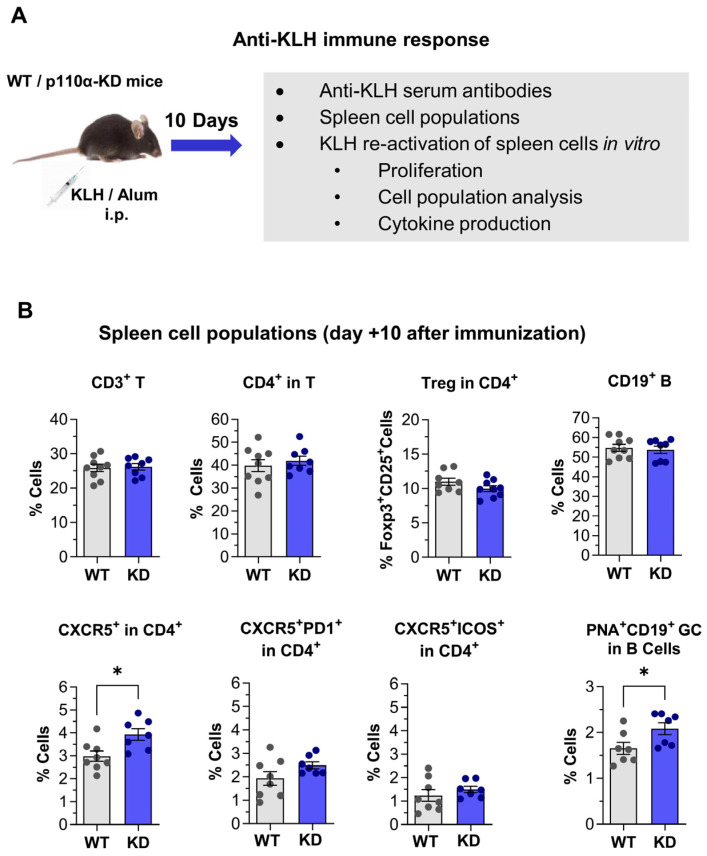
Effect of T cell-specific inactivation of PI3-kinase p110α in the primary immune response against the keyhole limpet hemocyanin (KLH) protein antigen. (**A**) Outline of experimental procedures. WT (*n* = 9) or p110αKD-T (KD, *n* = 8) mice were injected intraperitoneal (i.p.) with KLH in Alum. (**B**) Lymphocyte subpopulations in the spleen of WT (grey) or p110αKD-T (KD, blue) immunized mice, 10 days after KLH injection. Top panels, percentage of CD3^+^ (T cells), CD4^+^ in T cells, Treg (FoxP3^+^CD25^+^) in CD4^+^ cells and CD19^+^ B cells; bottom panels, percentage of CXCR5^+^ cells, CXCR5^+^PD-1^+^, or CXCR5^+^ICOS^+^ in CD4^+^ cells, and germinal center CD19^+^PNA^+^ in B cells. Data from individual mice and the mean ± SEM for each group are shown. Significant differences between WT and KD mice were determined by the Student’s *t* test (* *p* < 0.05).

In view of the above data, the specific *in vivo* response to a protein antigen (keyhole limpet hemocyanin, KLH) was studied in the p110αKD-T model (Figure 8A).

**Figure 9:**
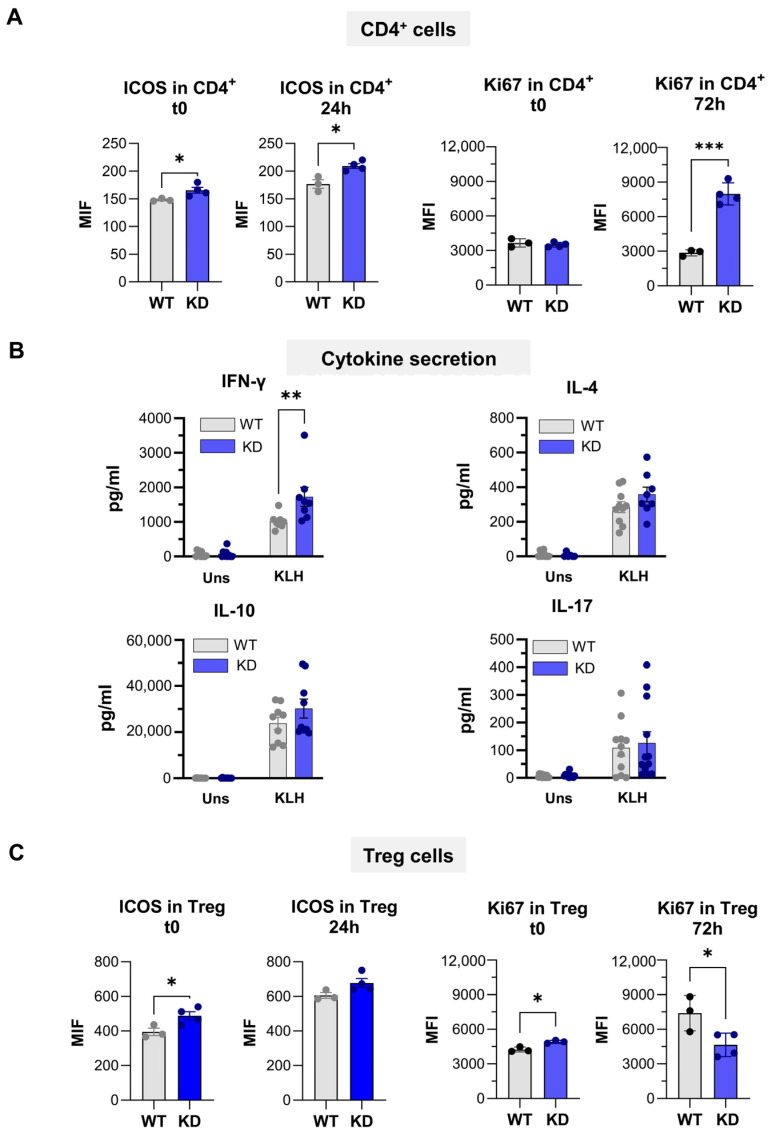
Antigen-specific in vitro re-stimulation of T cells from KLH-immunized WT (grey) or p110αKD-T (KD, blue) mice. (**A**) Expression of markers of activation (ICOS, left panels) or proliferation (Ki67, right panels) were determined in spleen cells from individual mice before (t = 0) or after culture for 24 h (ICOS) or 72 h (Ki67) in the presence (KLH) or absence (Uns) of added KLH. (**B**) Cells from individual mice were cultured with (KLH) or without (Uns) the KLH antigen. At 72 h, supernatants were taken, and cytokines (IFN-γ, IL-4, IL-10, and IL-17) were assessed. (**C**) Expression of functional (ICOS, left panels) and proliferation (Ki67, right panels) markers in CD4^+^CD25^+^Foxp3^+^ Treg spleen cells from individual mice before (t = 0) or after culture for 72 h in the presence of KLH. Significant differences between WT and KD mice determined by the Student’s *t* test are shown (* *p* < 0.05, ** *p* < 0.01, *** *p* < 0.001).

Ten days after immunization, the proportion of germinal center B cells and CXCR5^+^ CD4^+^ T cells was significantly increased in p110αKD-T mice, but CXCR5^+^ICOS^+^ or CXCR5^+^PD-1^+^ Tfh cells were not significantly enhanced in mutant mice (Figure 8B). Intriguingly, the level of ICOS expression in p110αKD-T CD4^+^ cells from immunized mice was increased (Figure 9A, left panel).

Antigen-specific activation in vitro induced higher levels of ICOS or the proliferation marker Ki67 in KLH-immunized p110αKD-T CD4^+^ than in WT cells (Figure 9A). Both types of cells equally responded to polyclonal activation by anti-CD3 antibody in terms of Ki67 expression, but again, ICOS expression was higher in p110αKD-T CD4^+^ cells (Supplementary Figure S8B).

In KLH-immunized p110αKD-T mice, the secretion of IFN-γ was significantly higher upon antigen re-stimulation in vitro (Figure 9B). No significant differences were observed in the secretion of other relevant cytokines like IL-4, IL-10, or IL-17A upon activation with KLH antigen (Figure 9B).

When CD4^+^ Treg cells were studied in the KLH-immunized p110αKD-T mice, enhanced ICOS expression levels were observed in Treg cells (p110aKD vs. WT) either freshly obtained (Figure 9C, t0) or after anti-CD3-in vitro stimulation of the cells (Figure S8B). In addition, lower Ki67 expression in p110αKD Treg cells from KLH-immunized mice upon in vitro re-stimulation with KLH may denote a defect mediated by p110α in the antigen-induced proliferation mechanism (Figure 9C and Figure S8).

Last, the effect of the T cell-specific inactivation of p110α in the production of KLH-specific antibodies was analyzed (Figure S8C). Despite the differences observed in relevant functional populations and markers, as well as in cytokine production, WT or p110αKD-T mice did not show significant differences in the titer of anti-KLH antibodies, including antibodies of the IgM, IgG1, IgG2b, and IgG2c subclasses, at the time considered.

## 3. Discussion

The role of the class IA p110α isoform of PI3K in T cell development and function was studied using a model of T cell-specific inactivation in vivo (p110αKD-T). Our data showed a significant role for p110α in T lymphocyte differentiation in the thymus, particularly in the CD4^+^CD8^+^ DP T cells, CD4^+^ SP T cells, and CD4^+^ SP Treg populations, whereas differences in T cell differentiation, homeostasis, and function in the periphery were mainly observed in CD4^+^ T cells, the Tfh, and Treg populations.

### 3.1. Thymus Differentiation

The thymus of p110αKD-T mice had increased cellularity that was mainly due to an enhanced population of the immature thymus CD4^+^CD8^+^ DP population, which makes up the highest proportion in this organ. Since p110α inactivation is specific to CD4^+^ cells, we observed no effect in the immature DN cells or their different developmental stages. The analysis of different developmental steps within CD4^+^CD8^+^ DP cells showed no differences except for a significantly higher apoptosis in the late CD3^high^CD5^high^ DP3 thymocytes and lower apoptosis in early immature (M1) CD3^high^CD24^high^ CD4^+^ SP stages. This suggests that the excess cell number is partially corrected in the negative selection process. In contrast, our previous data in a model of T cell-specific deletion of p110α (p110α ΔT model) showed no significant differences in thymus cell numbers or subpopulations ([17]). The number of cells per spleen was significantly increased in p110αKD-T mice, whereas our previous data in mice with T-cell specific removal of p110α showed a lower number of cells and lower percentage of CD4^+^ T cells in the spleen [17].

Our data showing enhanced Erk activation in p110αKD-T thymocytes fit with the importance of Erk activation in the positive selection of DP thymocytes and in the lineage commitment of CD4^+^ SP [38,39]. Furthermore, enhanced Akt activity could favor tonic and antigen-induced TCR signals and thymocyte survival [40,41], while blocking the generation of thymus-derived Treg cells (reviewed in [42]).

Taken together, the data suggest an enhanced positive selection in p110αKD-T thymus that is partially corrected in the mature DP population that undergoes negative selection. The enhanced apoptosis in the final steps of DP maturation only partially corrects the excess CD4^+^ SP, which is still higher in p110αKD-T mice.

Previous data using gene inactivation, the expression of kinase-dead isoforms, and/or isoform-specific inhibitors showed that both class IA p110δ and class IB p110γ PI3K subunits were essential to T cell differentiation in the thymus during both pre-TCR- and TCR-dependent steps [12,43,44,45,46,47,48]. In particular, double negative p110γδ^−/−^ or p110γ^−/−^δ^DA/DA^ mice had thymuses with markedly reduced size and cellularity that was mainly due to a low number and increased apoptosis of DP thymocytes [12,46]. The difference between p110α and p110δ inactivation suggests a dominant role of p110δ signals in protecting thymocytes from apoptosis at the DP steps, which is attenuated in the presence of functional p110α.

### 3.2. PI3K in Treg In Vitro and In Vivo

Class I PI3Ks have a complex role in Treg development and homeostasis in vivo and in vitro [6,49]. Thus, whereas p110δ inactivation can enhance Treg differentiation in the thymus linked to lower PI3K/Akt/Foxo1-mediated inhibition of Foxp3 expression, it can also impair IL-2-CD25 PI3K signaling leading to a reduced Treg proportion and function in the periphery [20,50,51]. The overall impact of these effects on immune responses can be paradoxical, i.e., although p110δ inactivation in mice causes deficient T cell effector responses, the stronger functional impairment of Tregs caused by p110δ inactivation leads to protection against different types of cancer, and this effect can be mimicked by the administration of p110δ-specific inhibitors [21,52].

Unlike p110δ inactivation, we showed here that p110α inactivation in T cells impaired Treg development in the thymus. Furthermore, the proportion of Treg in the spleen remained lower in p110αKD-T mice, and Treg differentiation in vitro was inhibited. In contrast, a time-dependent enhancement of Treg differentiation has been reported in vitro upon PI3K inhibition, which is particularly dependent on p110α [53].

Like p110α inactivation, our previous data in a model of T cell-specific deletion of p110α (p110α ΔT) also indicated lower proliferation of T cells under Treg differentiation conditions in vitro and a lower percentage of Treg cells in draining lymph nodes during anti-tumor responses [17]. Treg-specific deletion of PI3K isoforms show no significant effect of p110α deletion and confirms the major role of p110δ in normal Treg differentiation and function [25]. Yet, deletion of both isoforms leads to a profound loss of Treg cells in vitro, as well as the increased severity of experimental autoimmune encephalitis and spontaneous development of neuronal inflammation [25].

The deletion of p110α differentially affects the homeostasis of CD4^+^ spleen populations, as p110α ΔT mice have a lower percentage of total and naive CD4^+^ T cells, particularly in older mice [17]. Interestingly, the percentage of Treg cells and the ratio of Treg to total CD4^+^ cells is enhanced over time, suggesting that Treg cells are less dependent on p110α than other CD4^+^ cells for long-term survival in the periphery [28]. Moreover, these changes affect the sensitivity of mice to autoimmune encephalitis [28].

### 3.3. Tfh Generation In Vitro and In Vivo

Intriguingly, p110αKD-T mice immunized with protein antigen had enhanced CD4^+^ cells expressing the CXCR5 receptor needed to reach germinal centers (Figure 8), yet bona fide Tfh cells expressing both CXCR5 and ICOS or PD1 [17,54] were not significantly enhanced in immunized p110αKD-T mice (Figure 8). The differentiation of Tfh in vitro was lower in p110αKD-T mice (Figure 7), and p110α inhibition showed that unlike other cytokines, IL-21 production by cultured CD4^+^ T cells was dependent on p110α [16]. In fact, when IL-21 was provided to the cultures during differentiation, p110αKD-T cell proliferation was higher than WT proliferation (Figure S7A). Taken together, these data indicate that the inactivation or inhibition of p110α hampers full Tfh differentiation, but this can be at least partially overcome by additional stimuli. Previous data from CD4^+^ cells lacking p110α (p110α ΔT) showed enhanced differentiation of Tfh in vitro and an enhanced proportion of Tfh cells in vivo [17,54].

Interestingly, germinal center B lymphocytes were significantly enhanced in both p110α KD-T and p110α ΔT immunized mice (Figure 8, [17,54]), yet antibody responses showed no significant changes in p110αKD-T (Supplementary Figure S8) but were enhanced in p110α ΔT mice [17,54]. One reason for these differences might be that in p110αKD-T mice, B cells are retained in the germinal centers, but Tfh do not efficiently promote the progression of B cells into antibody-producing cells.

Some of the functional differences observed in P110αKD-T vs. P110α ΔT might stem from differences in downstream signaling pathways triggered by antigen activation, since MAP kinase and PI3K/Akt/mTOR pathways have complex interactions (see, e.g., [55,56]). PI3K catalytic subunits p110α have stronger interactions with PI3K regulatory subunits than p110δ [26] and with different Ras family activators [57]. Thus, the deletion of one PI3K subunit can alter signaling differently from inactivation or inhibition. Previous data with inhibitors of p110α [17,26,28] or inactivation in the p110αKD-T model (this paper) showed lower Akt, ERK, or NFAT activation in Tfh and other mature T helper cells. On the other hand, p110α deletion or silencing in T cells consistently leads to enhanced ERK activation and lower Akt activation. Intriguingly, unlike mature T cells, we showed that early activation of Erk and Akt was enhanced in thymocytes from p110αKD-T mice. Also, distinct signaling characteristics might be behind the different outcomes between p110αKD-T and p110α ΔT mice in terms of cytokine production by T cells, namely, no effect or inhibition in most cases in p110αKD-T mice but enhanced cytokine secretion by cells from p110α ΔT mice.

Thus, T cell-specific PI3K p110α inactivation alters T cell differentiation and function and gives some clues as to the T cell-specific effects of p110α-inhibitors that are of interest in anticancer therapies or in autoimmune disease. The long-term impact of T cell-specific p110α inactivation in the evolution of immunological memory and aging, or in distinct autoimmune and anticancer responses, and the possibility of using therapies directed at specific cell lineages deserve further analysis.

## 4. Materials and Methods

### 4.1. Mice

CD4-Cre mice (strain B6;D2-Tg(Cd4-cre) 1Cwi/CwiCnrm, [58]), *Pik3caflox*, p110α^flox/flox^ mice [59], and mice with T cells deficient for PI3K p110α subunits (CD4-Cre^+/–^ p110α^flox/flox^; p110αΔT) [17,27,28], all in a C57BL/6J background, have been previously described. Mice with a conditional kinase dead mutation in the catalytic domain (p110α cKD) were generated at CNIO (Madrid, Spain). A minigene containing exons 18–20 of p110α followed by a transcription STOP sequence and an inverted PGK-neo cassette, all flanked by loxP sites, was inserted in intron 17 of the p110α gene by DNA homologous recombination in ES cells. Simultaneously, a D933A mutation was introduced in exon 19 of the endogenous gene to inactivate the ATP-binding motif of p110α, (See Figure S9). The minigene was designed so that in the absence of Cre, the WT form of p110α was expressed from the targeted allele. Cre-mediated deletion of the floxed sequence allowed the expression of the kinase-dead p110α mutant form. The line was maintained in heterozygosity by backcross with C57BL/6J mice (p110α^wt/KDflox^ mice) in the Animal Facility of Instituto de Salud Carlos III. To obtain mice with T cell-specific inactivation of p110α (p110αKD-T mice), p110α^wt/KDflox^ mice were crossed with CD4-Cre homozygotes in a homozygous *Pik3caflox* background (CD4-Cre^+/+^ p110α^flox/flox^ mice, CD4-Cre^+/+^ p110α ΔT). Age- and sex-matched littermates with inactivated PI3K p110α in T cells (CD4-Cre^+/–^ p110α^Δ/KD^; p110αKD-T) and their Cre^+/–^ littermates (CD4-Cre^+/–^ p110α^Δ/wt^; WT) were used throughout the experiments. All mice were bred in the animal care facility of Instituto de Salud Carlos III (Majadahonda, Madrid, Spain) under specific opportunistic and pathogen-free conditions from stock obtained from the animal facility of CNIO, or purchased from Charles River laboratories (Écully, France) or the European Mouse Mutant Archive (EMMA, GMBh; CD4-Cre). All procedures were conducted under project licenses PROEX 330/15 and PROEX 401.8/21 (P.P., ISCIII) and PROEX 204.8/20 (S.O., CNIO), respectively, issued by the Consejería de Medio Ambiente y Ordenación del Territorio, C.A. Madrid, Spain, and complied with institutional, national and European Union ethical and animal welfare guidelines.

### 4.2. Genotyping

To genotype p110αKD-T littermates, genomic DNA from heparinized blood was extracted, and target amplification was performed by direct PCR using the REDExtract-N-Amp^TM^ Blood PCR Kit (Sigma-Aldrich-Merck, Darmstadt, Germany; Ref. XNAB) following the manufacturer instructions, in a 2720 Thermal Cycler (Applied BioSystems). The following oligonucleotides were used to detect the allele containing the lox-flanked minigene Pik3ca^LmLD933A^ (Lml, lox-minigene-lox):

Pik3ca LmLD933A Gentp1 5′-FW: GCTCTTGGGCCTCCACAGGTG

Pik3ca LmLD933A Gentp2 5′-RV: ACACGTTCCCGCTTATAGCC

For genotyping parental p110α^wt/KDflox^, genomic DNA was extracted from a biopsy of ear tissue, and direct PCR was performed using the REDExtract-N-Amp kit (Sigma-Aldrich; Merck Spain; Ref. XNAT) following the manufacturer instructions. Pik3ca LmLD933A Gentp1 5’-FW and Pik3ca LmLD933A Gentp2 5’-RV oligonucleotides were used.

For genotyping parental CD4-Cre^+/+^ p110α^flox/flox^ mice, q-PCR was performed by using the PowerUp^TM^ SYBRT^M^ Green Master Mix ((Applied BioSystems-ThermoFisher Scientific, Waltham, MA, USA) Ref. A25742), following manufacturer instructions, in a Quant Studio QS3 Real-Time PCR machine (ThermoFisher Scientific). The β-tubulin gene was used as an internal control. DNA from heterozygous Cre^+/−^ mouse was used as a reference to select homozygous mice in the littermates. The following oligonucleotides were used:

Cre 5′FW: GATGCAACGAGTGATGAGGT

Cre 5′RV: GCATTGCTGTCACTTGGTCGT

β-tubulin 5′-FW: GCCAGAGTGGTGCAGGAAATA

β-tubulin 5′RV: TCACCACGTCCAGGACAGAGT

### 4.3. Antibodies and Cytokines

Antibodies used in tissue culture, flow cytometry, Ig ELISA, and immunoblot are listed in Table S1. Recombinant cytokines were from Peprotech-ThermoFisher Scientific (human IL-2 and mouse IL-4, IL-6, IL-12 and IL-21) or R&D (human TGF-β).

### 4.4. Analysis of Surface and Intracellular Markers by Flow Cytometry

Single-cell suspensions were incubated for 15 min with 10% heat-inactivated normal mouse serum in ice-cold staining buffer (PBS with 2% heat-inactivated FBS) to block Fc receptors and then with the indicated labelled antibodies to detect cell-surface molecules; after extensive washing, they were analyzed by flow cytometry, gating lymphoid cells according to FSC/SSC. Fluorochrome-coupled antibodies against the selected surface antigens and their appropriate isotype controls are shown in Table S1. To detect apoptotic cells, Annexin V-FITC apoptosis detection kit (Inmunostep S.L., Salamanca, Spain) was used. Data were acquired in a FACS LSR Fortessa^TM^ X-20 (BD Biosciences, Franklin Lakes, NJ, USA) flow cytometer and analyzed by using FACSDiva^TM^ v 8.0.1 (BD Biosciences) or FlowJo software (Tree Star, Inc., Ashland, OR, USA, Version 10.0).

For the intracellular staining of FoxP3 (to detect Treg) or Ki67 (as proliferation marker), cells were first surface-stained with the necessary antibodies as above, and then they were suspended, washed, fixed, and permeabilized with the Transcription Factor Staining Buffer Set (eBioscience-ThermoFisher Scientific) as indicated by the manufacturer. Then, the cells were stained with anti FoxP3-PE (clone 3G3, eBioscience), KI67 (clone SolA15), or a control isotype antibody according to the protocol for intracellular staining of transcription factors. Data were then acquired on a LSR Fortessa^TM^ X-20 (BD Biosciences) flow cytometer and analyzed as described above.

### 4.5. Differentiation of Helper and Regulatory CD4^+^ T Cells “In Vitro”

Splenic naïve T lymphocytes from p110αKD-T mice or control littermates were differentiated in vitro into Th1, Th17, Tfh, or Treg cells as described previously [17,27]. Briefly, CD4^+^CD62L^+^ T lymphocytes were isolated from spleen cell suspensions using the CD4^+^ T-cell isolation kit II (130-090-860, Miltenyi Biotech, Bergisch Gladbach, Germany). After washing, the cells were cultured at 10^6^ cells/mL in 24- or 48-well culture plates (Costar 3524, 1 mL/well, or Costar 3548, 0.5 mL/well) that were pre-coated with anti-CD3 antibodies (YCD3-1, 5 µg/mL [60]) and specific antibodies and cytokines were added. Th1 differentiation was carried out for 72 h in the presence of anti-CD28 antibody (2.5 µg/mL), anti-IL-4 (5 µg/mL), and IL-12 (20 ng/mL). Th17 differentiation was performed in the presence of anti-CD28 (2.5 µg/mL), anti-IL-4 (5 µg/mL), and anti-IFN-γ (10 µg/mL) plus IL-6 (20 ng/mL) and 5 ng/mL human TGF-β1 (5 ng/mL); Tfh differentiation was similar, except that TGF-β1 was omitted. For Treg differentiation, cells were cultured 48 h in anti-CD3-coated wells in the presence of anti-CD28 antibody (1 µg/mL), anti-IL-4 (5 µg/mL), anti-IFN-γ (10 µg/mL), 20 ng/mL human IL-2, and 5 ng/mL TGF-β1.

### 4.6. Early Activation Signals in T Cells

Intracellular signals were determined as described in detail in [17,27]. Single-cell suspensions of thymus cells were thoroughly washed. Then, the cells (4 × 10^7^ cells/mL in serum-free medium) were mixed 1:1 with latex beads previously coated with anti-CD3 (2C11, 5 µg/mL) or a control antibody. Furthermore, anti-CD3 activation was carried out in the presence of 10 µg/mL of anti-CD28 antibody. Activation was stopped after 10 min of incubation at 37 °C.

After washing with ice-cold PBS, 500 µM EDTA, 200 µM NaVO_4_, the cells were lysed for 15 min on ice with 1% Triton X-100 (1% Triton X-100 in 50 mM Tris/HCl, 150 mM NaCl, pH 7.6, 1 mM MgCl_2_, 1 mM EGTA, 1 mM NaVO_4_, and a protease inhibitor cocktail (Selleckchem, Houston, Texas, USA) at 4 × 10^7^ cells/mL. Post-nuclear lysates were mixed 3V:V with a 4× SDS-PAGE sample buffer and separated in 10% acrylamide SDS–PAGE.

For the experiments described in Figure 7C, CD4^+^ naïve spleen T cells (10^6^/mL) were activated for 5 days in the presence of mitomycin C-treated APC (10^6^/mL) and 2.5 µg/mL Concanavalin A in the presence of 10 ng/mL IL-21 to favor Tfh differentiation [61]. Cells were washed and rested for 2 h at 37 °C before activation with anti-CD3 plus anti-CD28 as described above. Blast cell lysis was performed at 2 × 10^7^ cells /mL, and post-nuclear lysates mixed V:V with a 2× SDS-PAGE sample buffer.

Phosphorylated proteins and loading controls were analyzed by immunoblot as described in [17,27].

### 4.7. Elongation Assay

ICOS-induced elongation of CD4^+^ T cells was determined as described in [17] using Tfh cells differentiated in vitro that expressed high levels of ICOS (see Section 4.5). Elongation assays were performed in µ-Slide 8-Well Glass Bottom slides (Ibidi GmbH, Gräfelfing, Germany) previously coated with anti-ICOS antibody or Poly-L-Lysine (10 µg/mL in PBS) [26]. After washing with PBS, the wells received 10^5^ cells in 0.2 mL of PBS, 10 mM HEPES, 0.1% glucose, and pH 7.2. The slides were briefly spun and then incubated (60 min at 37 °C in 5% CO_2_ atmosphere). After washing with PBS, they were fixed with 1% formaldehyde. The cells were analyzed in an inverted optic microscope (Leica DMI3000 B) with a Leica DFC 420 CCD camera at a 40× magnification to acquire images, with LAS v4.13 Software (Leica, Wetzlar, Germany).

Cells were eventually analyzed with the shape descriptors of the ImageJ 1.51j8 public domain software (National Institutes of Health, Bethesda, MD, USA); the elongation (axis ratio) was determined as the ratio of the major and minor axis of the ellipse fitting each cell.

### 4.8. Cytokine ELISA

Cytokines IL-2, IL-4, IL-6, IL-10, IL-17A, IL-21, IFN-γ, and TNF-α were determined by capture ELISA using kits from eBioscience (Ready-SET-Go! Kits, eBioscience; San Diego, CA, USA).

### 4.9. Immunization of p110αKD T Mice with KLH

Mice were injected i.p. with 100 µg of keyhole limpet hemocyanin (KLH, Sigma-Aldrich-Merck; Ref. 374825) in Alum. On day 10 after immunization, the mice were euthanized before obtaining lymphoid organs or blood by cardiac puncture.

### 4.10. Anti-KLH Serum Antibodies

Anti-KLH antibodies were assessed in the serum from immunized mice by ELISA. Wells in 96-well ELISA plates (Costar-ThermoFisher Scientific) were first coated with 20 µg/mL KLH in PBS then incubated with serial dilutions of serum and eventually with horseradish peroxidase-coupled antibodies specific to the mouse Ig subclasses IgM, IgG1, IgG2b, and IgG2c, all from Southern Biotech (Birmingham, AL, USA). Titer was considered as the reciprocal of the serum dilution yielding 50% of the maximal optical density.

### 4.11. KLH-Specific Responses In Vitro: Assessment of Cell Proliferation and Cytokine Secretion

Cell suspensions obtained from the spleens were used to determine T and B lymphocyte subpopulations. The in vitro activation of spleen cells from immunized mice with KLH antigen was carried out in 48-well culture plates (Costar-ThermoFisher Scientific). Spleen cells (10^6^/culture in 1 mL of culture medium) were incubated for 72 h in the presence of 100 µg KLH. Then, cultures were resuspended, centrifuged, and the supernatants were taken to determine secreted cytokines by ELISA. Cells in the cultures were then processed to determine surface markers, intracytoplasmic cytokines, or proliferation (Ki67) by flow cytometry. Cultures without antigen or with anti-CD3 (YCD3-1 [60], obtained in-house, 2 µg/mL) were used as negative and positive activation controls, respectively.

### 4.12. Statistical Analyses

Data were analyzed by using the GraphPad Prism 10 software (GraphPad Prism Software Inc., La Jolla, CA, USA), and they are shown as the mean ± standard error of the mean (SEM). Data from biological replicates (mice) were analyzed individually. Direct group–group comparisons were assessed by using Student’s *t*-test test for unpaired, two-tailed experimental design. A one-way ANOVA was used when multiple comparisons were necessary. Significant differences between data are indicated by asterisks (* *p* < 0.05, ** *p* < 0.01 and *** *p* < 0.001). All statistical calculations refer to the control group or as indicated by brackets.

## 5. Conclusions

Class IA PI3 kinases are targets for immunomodulation as major players in normal and pathological immune responses and inflammation. In T lymphocytes, they mediate signals delivered by different receptors, including antigen receptors and costimulatory molecules like CD28 and ICOS. Since PI3K p110α is expressed at high levels in T cells, it is of interest to know its potential in immunomodulation. Furthermore, we need to know the effect on immune parameters of PI3K inhibitors with broad or narrow specificity, of interest in cancer, metabolism, or autoimmunity.

Genetic models complement studies on the effect of specific inhibitors and thus, using a conditional transgenic mouse model, we showed here the implication of PI3K-p110α in the intracell signaling and differentiation of thymocytes, particularly in the DP and SP CD4^+^ stages and in the differentiation of thymic Tregs. The effect of p110α inactivation extended to different functional phenotypes within the CD4^+^ population of the spleen, as shown in in vitro differentiation experiments of induced Tfh and Treg cells and in primary response assays to Ag in vivo and recall antigen activation in vitro.

Understanding the implication of PI3K isoforms in the molecular mechanisms that regulate T cell differentiation and function will shed light not only in the physiology of the immune system but in the generation of autoimmune processes and the appearance of lymphomas. Our results with genetic models highlight the relevance of further studying the effect on the immune system of new, very specific p110α inhibitors approved for use in anticancer therapy [62]), which might be also beneficial in the therapy of autoimmune diseases. The ubiquitous expression of p110α in the organism suggests that therapy directed at specific cell lineages will open new, safer avenues in the application of PI3K p110α inhibitors.

## Data Availability

The data presented in this study are available from the corresponding authors on reasonable request.

## Supplementary Materials

The following supporting information can be downloaded at: https://www.mdpi.com/article/10.3390/ijms26020595/s1.
