# Supplementary material for: T Cell-Specific Inactivation of the PI3K p110α Catalytic Subunit: Effect in T Cell Differentiation and Antigen-Specific Responses
Source: Int J Mol Sci. 2025 Jan 12;26(2):595. doi: 10.3390/ijms26020595 (PMC11765243; doi:10.3390/ijms26020595)
Supplement: Supplementary file 1 [file ijms-26-00595-s001.zip › ijms-3422337-supplementary.pdf]

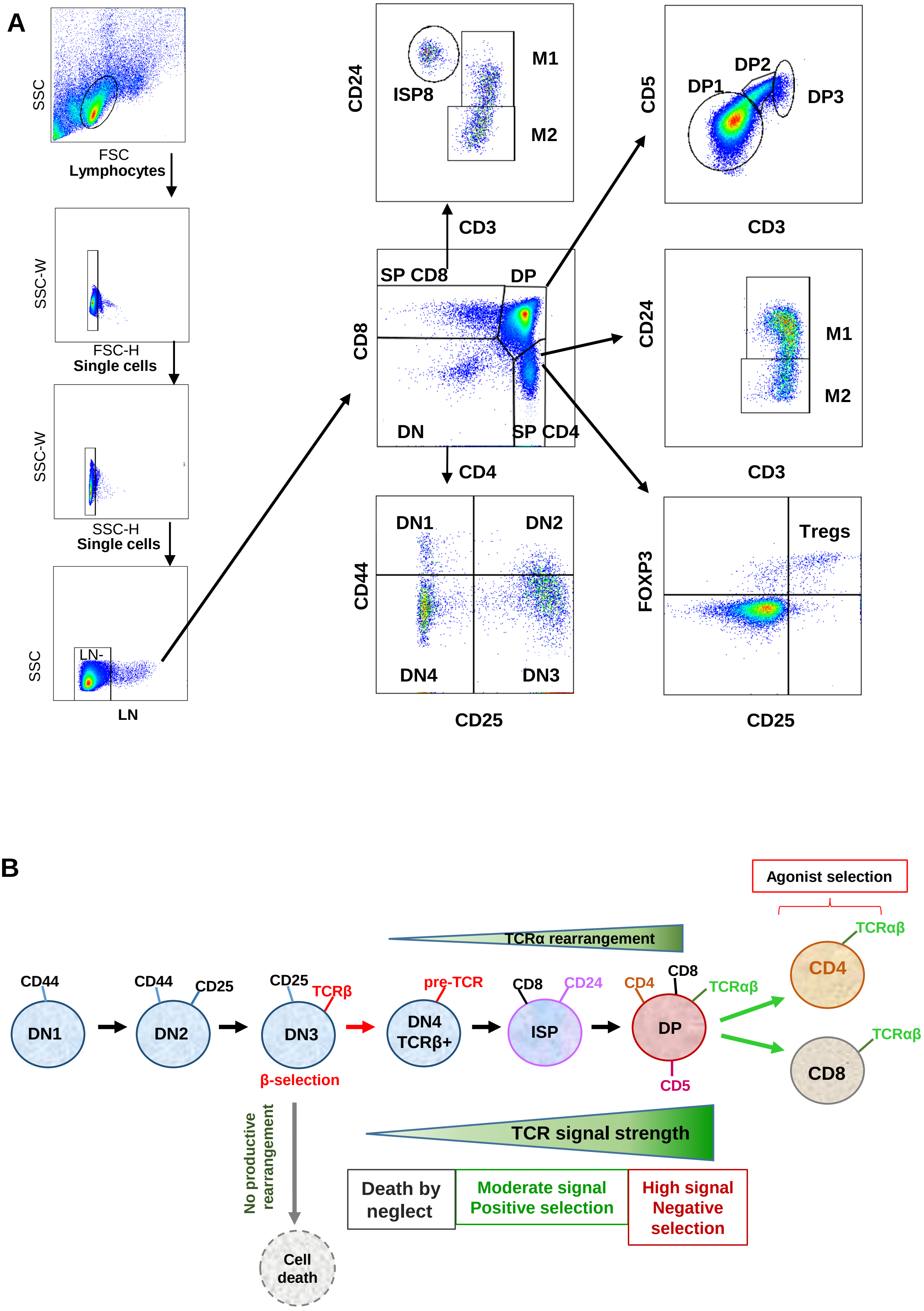
A
SSC
FSC
Lymphocytes
SSC-W
FSC-H
Single cells
SSC-W
SSC-H
Single cells
SSC
LN-
LN
CD24
ISP8
M1
M2
CD3
CD8
SP CD8
DP
DN
SP CD4
CD4
CD44
DN1
DN2
DN4
DN3
CD25
CD5
DP1
DP2
DP3
CD3
CD24
M1
M2
CD3
FOXP3
Tregs
CD25
B
Agonist selection
TCRα rearrangement
CD44
DN1
CD44
CD25
DN2
CD25
TCRβ
DN3
β-selection
pre-TCR
DN4
TCRβ+
CD8
CD24
ISP
CD4
CD8
TCRαβ
DP
CD5
TCRαβ
CD4
TCRαβ
CD8
No productive rearrangement
Cell death
TCR signal strength
Death by neglect
Moderate signal
Positive selection
High signal
Negative selection

Figure S1

Figure S1. Lymphocyte subpopulations during thymic differentiation. (A) Strategy for flow cytometry analysis of thymocyte subpopulation as defined by side scatter (SSC), forward scatter (FSC), lineage negative single cells (LN: $CD19^-$ $NK1.1^-$ $CD11c^-$ $TCR\gamma\delta^-$), CD4 and CD8 expression. Analysis for maturation stages in different thymus cells included $CD4^-CD8^-$ DN maturation stages DN1-DN4 defined by CD25 and CD44 expression; $CD4^+CD8^+$ DP maturation stages DP1-DP3 defined by CD3 and CD5 expression; $CD4^+CD8^-$ CD4 SP maturation stages M1 and M2, and $CD4^-CD8^+$ CD8 SP maturation stages M1, M2 and immature ISP8 as defined by CD3 and CD24 expression. Tregs within the $CD4^+CD8^-$ CD4 SP thymocytes were determined by CD25 and Foxp3 expression levels. **(B) Thymocyte differentiation, selection processes and cellular markers.** Three main differentiation stages in thymocytes are based on co-receptors CD4 and CD8 surface expression, defining double negative $CD4^-CD8^-$ (DN), double positive $CD4^+CD8^+$ (DP) and single positive (SP) $CD4^+$ or $CD8^+$. DN stage can be subdivided in DN1 to DN4 based on CD25 and CD44 expression together with TCRβ chain expression and pre-TCR (composed of TCRβ, pre-TCRα and CD3 subunits). The selection of productive TCRβ determines the survival or apoptosis of the thymocytes at DN3 stage and their progression to DN4 expressing a pre-TCR. Pre-TCR triggers rapid cell proliferation and progression to an intermediate $CD4^-CD8^+$ $CD24^{high}$ TCR^- ISP stage and subsequently into $CD4^+CD8^+$ DP. These cells remain quiescent for some days and latter undergo additional selection rounds where the TCR:MHC affinity is tested, dictating cell survival. DP subsets are based on cell surface CD3 and CD5 expression. The DP1 ($CD5^{low}CD3^{low}$) thymocytes give rise to DP2 ($CD5^{hi}CD3^{int}$), which differentiate to $CD4^+$ T cells. The DP3 ($CD5^{int}CD3^{hi}$) cells arise from DP2 and give rise to $CD8^+$ T cells. A small fraction of SP $CD4^+$ cells that show high TCR:MHC interaction (agonist selection) can be rescued from cell death to become Treg ($CD4^+$ $FoxP3^+$ $CD25^+$) or unconventional T cells (NKT, MAIT, etc). Eventually, SP $CD4^+$ and SP $CD8^+$ cells will exit the thymus to complete their maturation and differentiation in secondary lymphoid organs (30-36).

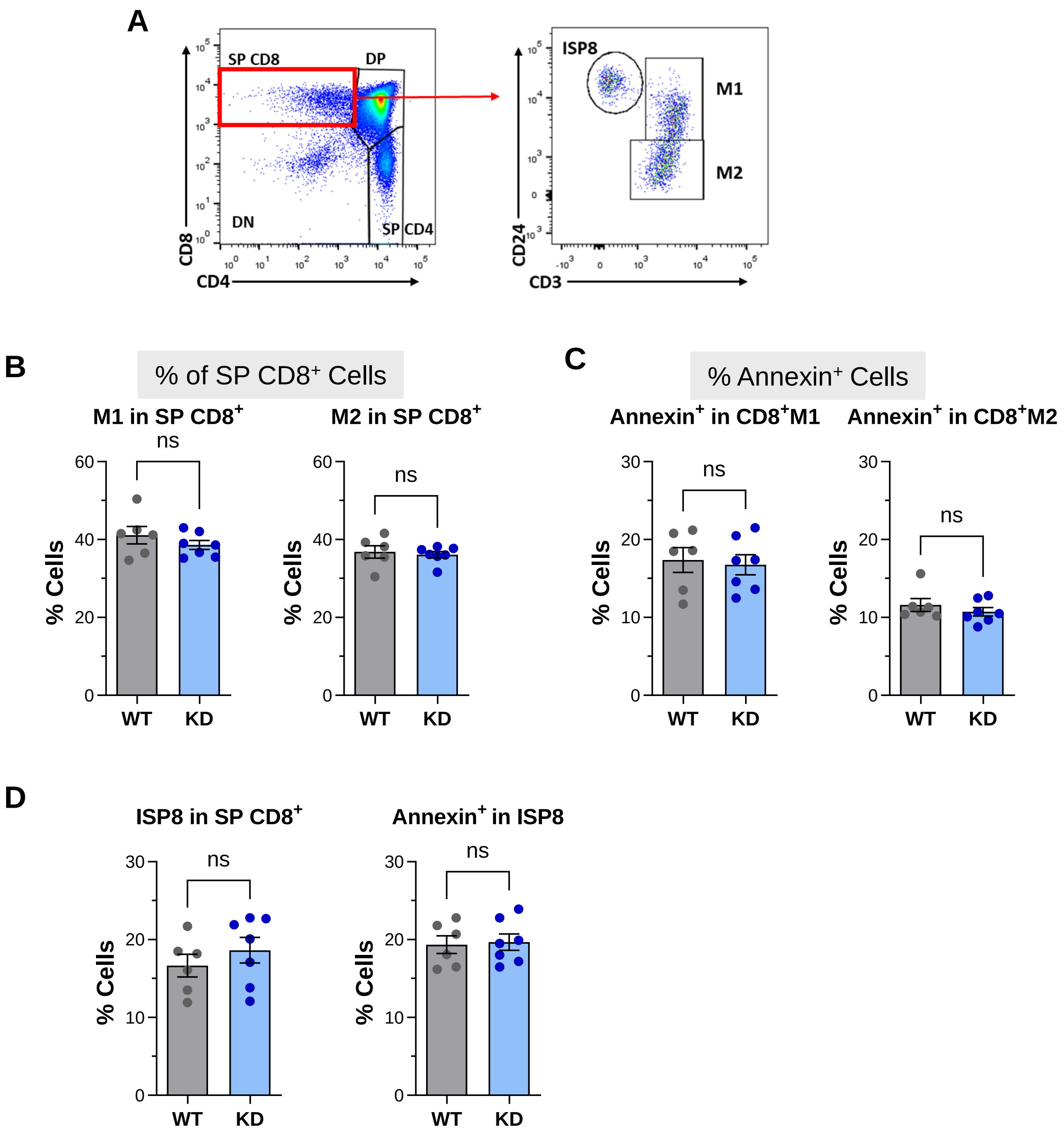

Figure S2. Maturation stages in WT and p110αKD-T SP CD8⁺ thymocytes. (**A**) Strategy for the analysis of $CD4^-$ $CD8^+$ SP $CD8^+$ thymocytes using CD3 and CD24 expression to determine the percentage of thymocytes in M1 and M2 maturation stages derived from $CD4^+CD8^+$ DP thymocytes, and the iSP8 population of SP $CD8^+$ thymocytes that are precursors of $CD4^+CD8^+$ DP cells. (**B**) Percentage of immature M1 (left) and mature M2 (right) SP $CD8^+$ thymocytes from WT (grey) or p110αKD-T mice (KD, blue). **C**) Annexin staining was used to assess the percentage of apoptotic cells within M1 (left) and M2 (right) $CD8^+$ SP thymocytes. (**D**) Percentage of iSP8 precursors ($CD8^+$ $CD3^{low}$ $CD24^{high}$) (left) and $Annexin^+$ apoptotic iSP8 cells (right) from WT (grey) or p110αKD-T mice (KD, blue). Data from individual WT (grey dots) or p110αKD-T mice (KD, blue dots) and the mean ± SEM for each group are shown. No significant differences were observed, as determined by the Student´s t test.

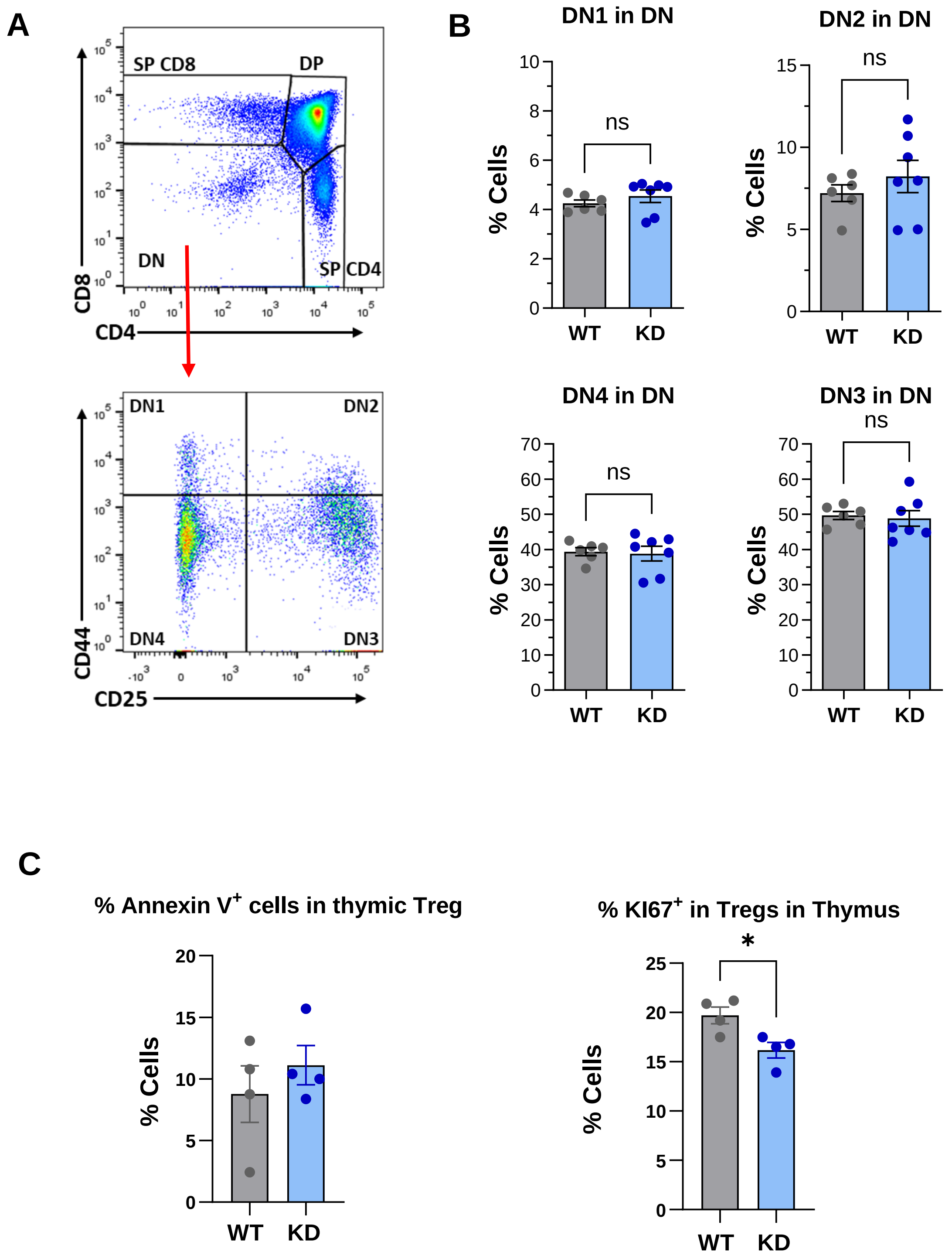

Figure S3. Analysis of thimic DN and Treg differentiation. A-B) Differentiation stages of WT and p110αKD-T $CD4^-CD8^-$ DN thymocytes, as determined by the expression of CD44 and CD25. (**A**) Dotplots showing the strategy used for the analysis of $CD4^-CD8^-$ DN thymus cells in the successive differentiation stages $CD44^{hi}CD25^-$ cells (DN1), $CD44^{hi}CD25^+$ cells (DN2), $CD44^{lo}CD25^+$ cells (DN3), and $CD44^{lo}CD25^-$ cells (DN4). (**B**). Percentage of $CD44^{hi}CD25^-$ DN1 cells (top left), $CD44^{hi}CD25^+$ DN2 cells (top right), $CD44^{lo}CD25^+$ DN3 cells (bottom right), and $CD44^{lo}CD25^-$ DN4 cells (bottom left), from WT (grey) or p110αKD-T mice (KD, blue). Data from individual WT (grey dots) or p110αKD-T mice (KD, blue dots) and the mean ± SEM for each group are also shown. No significant differences were observed between WT and KD cells, as determined by the Student´s t test.

C) Analysis of apoptosis (left) and proliferation (right) in thymic Treg cells. Cells were stained essentially as described in the Methods (section 4.4) but after surface labelling, the cells were treated with FITC-Annexin as described in 4.4 and then fixed/permeabilized to proceed with FoxP3 and Ki67 staining.

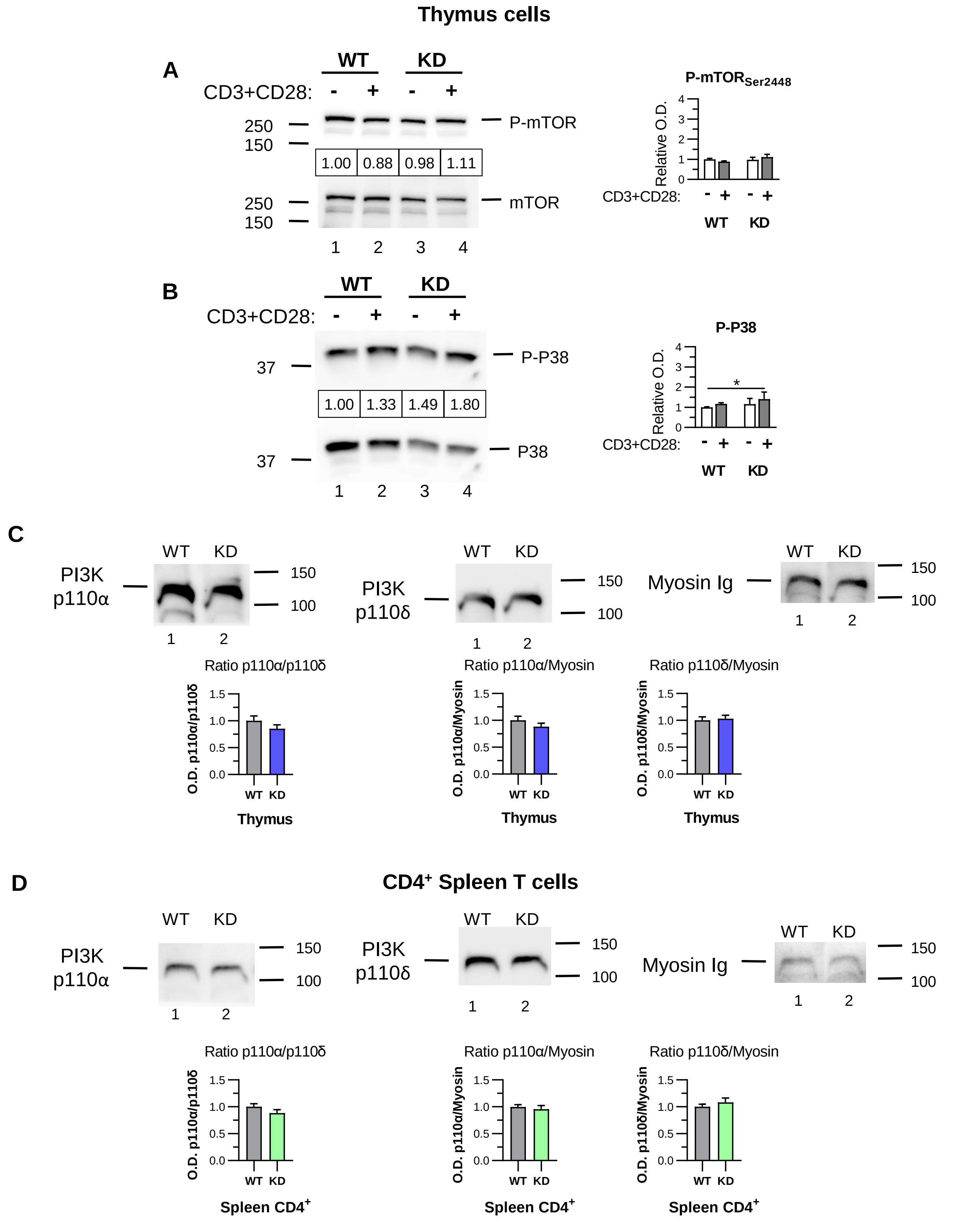

Figure S4.-***Cell signaling and PI3K expression in KD mice.***
(A,B) Thymus cells from WT and p110αKD mice were activated 10 min with anti-CD3 plus anti-CD28, or with control antibodies, as shown in the figure. Cell lysates were analyzed for mTOR$_{Ser2448}$ and P38$_{Thr180/Tyr182}$ MAP kinase phosphorylation by immunoblot. Significant differences between determinations are shown, as determined by one-way Anova (* $p < 0.05$). **(C)** Thymus cell lysates from WT and p110αKD mice were successively analyzed by immunoblot for expression of PI3K p110α and p110δ subunits, and then for the expression of Myosin Ig as a control protein. Graphs indicate the ratio of p110α to p110δ and the ratio of each subunit to the control protein. **(D)** Same as **(C)**, except that cell lysates from CD43+ naïve spleen T cells from WT and p110αKD mice were used. Asterisks show significant differences between WT and p110aKD-T cells, as determined by ANOVA (* $p < 0.05$).

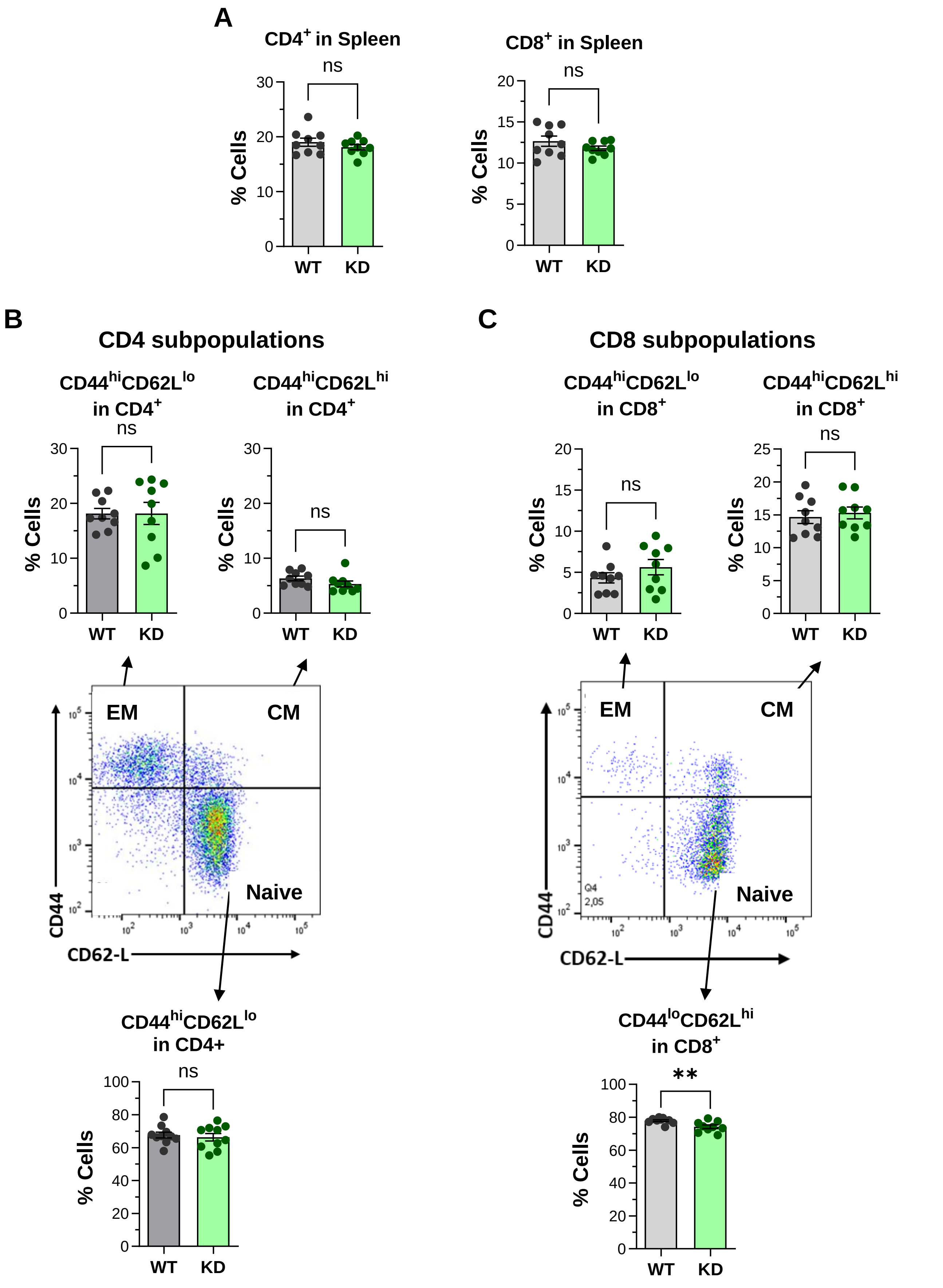

Figure S5. Flow cytometry analysis of $CD4^+$ and $CD8^+$ T cells effector subpopulations in the spleen of wild type mice (WT, grey) or p110αKD-T mice (KD, green). (**A**) Percentage of $CD4^+$ cells (left panel) and $CD8^+$ cells (right panel). (**B**) Representative flow cytometry histogram to assess the percentage of naïve $CD44^{lo}CD62L^+$, central memory (CM) $CD44^{hi}CD62L^+$, and effector memory (EM)$CD44^{hi}CD62L^-$ $CD4^+$ subpopulations from WT (grey) or p110αKD-T mice (KD, green), as indicated. (**C**) Representative flow cytometry histogram used to determine the percentage of naïve $CD44^{lo}CD62L^+$, central memory (CM) $CD44^{hi}CD62L^+$, and effector memory (EM) $CD44^{hi}CD62L^-$ $CD8^+$ subpopulations from WT (grey) or p110αKD-T mice (KD, green), as shown in the figure. Data from individual WT (grey dots) or p110αKD-T mice (KD, green dots) and the mean ± SEM for each group are also shown. Significant differences between WT and KD mice are depicted, as determined by the Student´s t test (** $p < 0.01$).

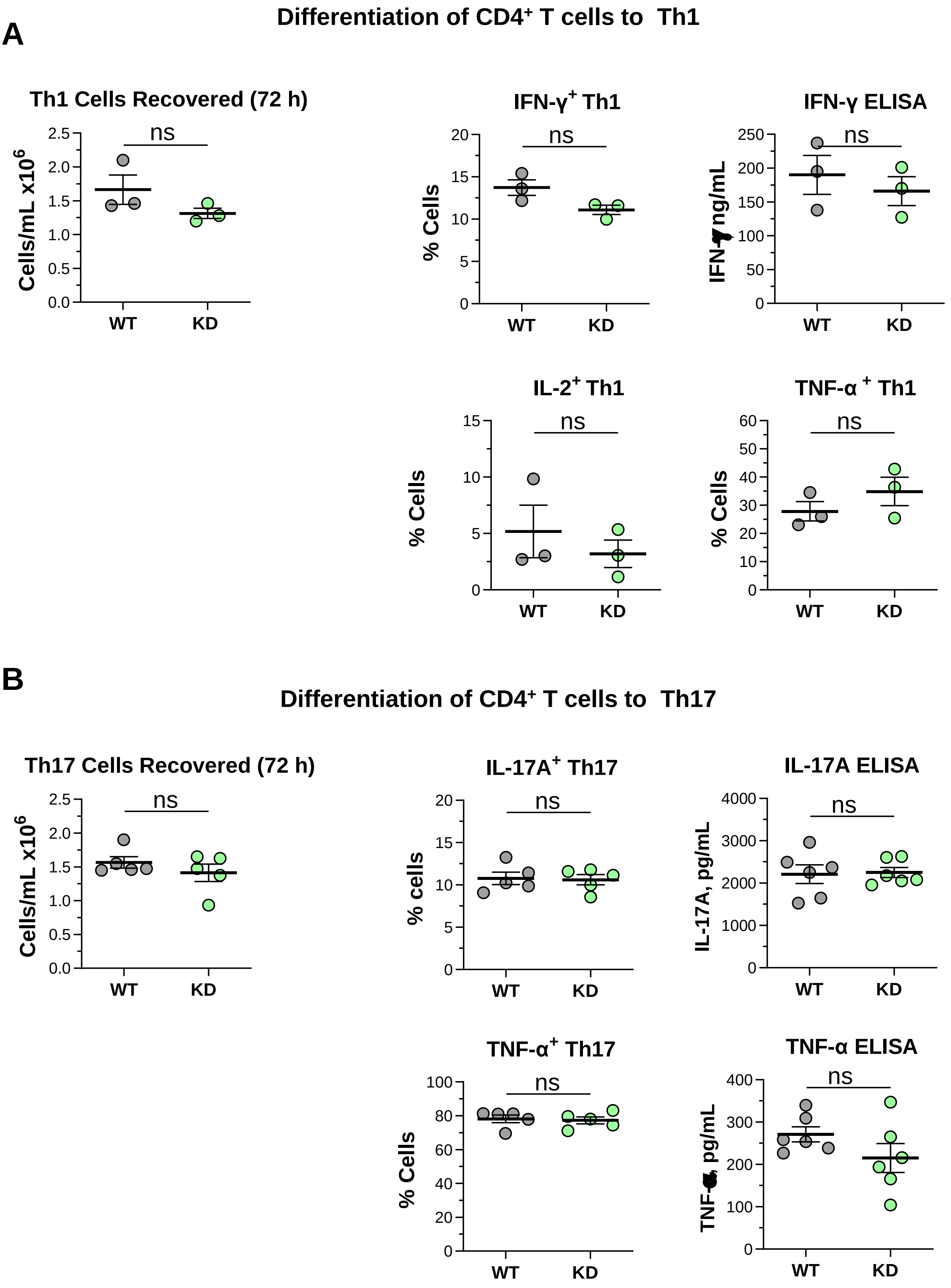

Figure S6. "In vitro" Th1 and Th17 differentiation of naïve CD4+ T lymphocytes from WT and p110αKD-T CD4+ T lymphocytes (grey and green circles, respectively). (**A**) Th1 differentiation was carried out using naive CD4+ T lymphocytes from WT or p110αKD-T cultured 72 h "in vitro" with anti-CD3 plus anti-CD28 antibodies in the presence of specific antibodies and cytokines (see Methods for details). Graphs show the number of cells recovered from 72 h culture (top left), the percentage of IFN-γ+ cells (center graph) and the concentration of IFN-γ in culture supernatants (right graph). Bottom panels show the percentage of IL-2+ and TNF-α+ cells, as indicated. (**B**) Th17 differentiation (see Methods for details). Graphs show the number of cells recovered from 72h cultures (top left), the percentage of IL-17+ cells (top center) and the concentration of IL-17 in culture supernatants (top right). Bottom panels show the percentage of TNF-α+ cells (left) and the concentration of TNF-α in culture supernatants (right). No significant differences were observed between WT and KD cultures, as determined by the Student´s t test.

Tfh blast cells

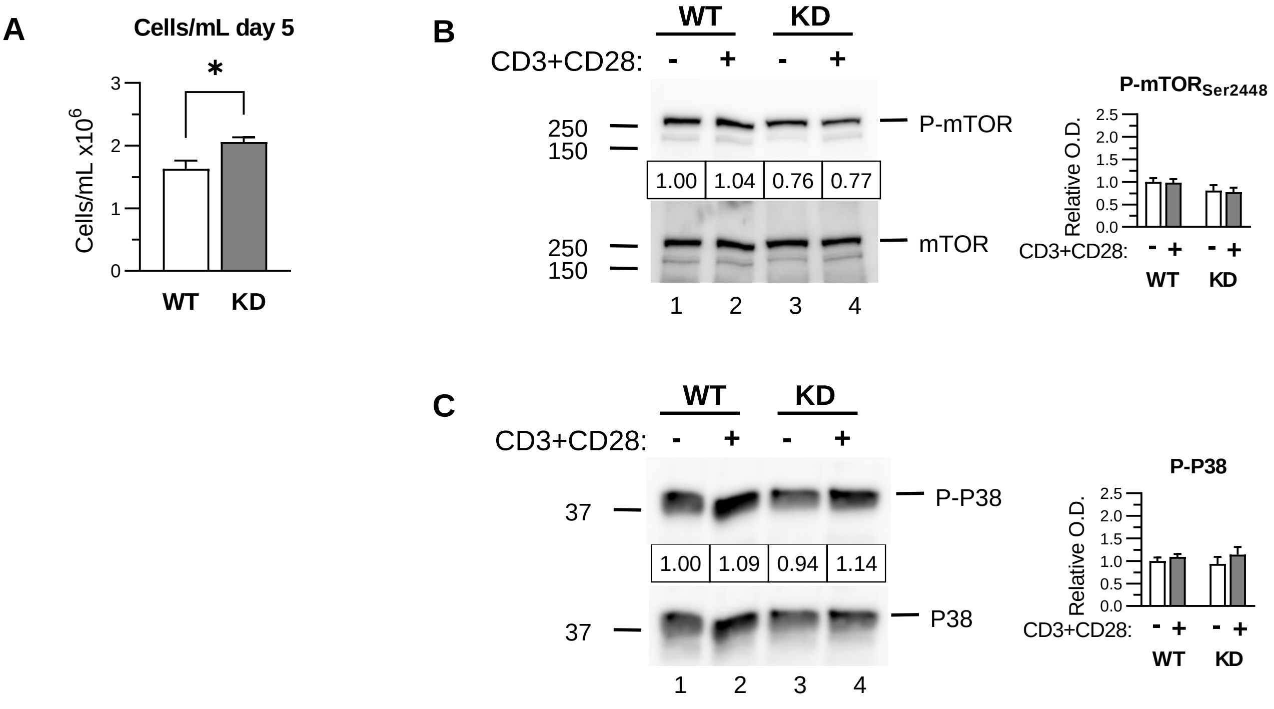

Figure S7.-***Differentiation of $CD4^+$ T cells into T follicular helper (Tfh) cells in vitro and T cell signalling.*** (**A**)) Tfh cell blasts were generated from naive $CD4^+$ T lymphocytes from WT (grey) or p110αKD-T (KD, green) cultured for 96 h “in vitro” with APC and Concanavalin A in the presence of IL-21. Cells recovered from cultures of WT or p110αKD-T (KD) cells are shown (WT n = 4, KD n = 3). Significant differences between WT and KD mice were determined by the Student´s t test (* $p < 0.05$). (**B,C**) $mTOR_{Ser2448}$ and $P38_{Thr180/Tyr182}$ MAP kinase phosphorylation in WT and p110αKD-$CD4^+$ T cell blasts activated 10 min with anti-CD3 plus anti-CD28, or with control antibodies, as shown in the figure. No significant differences were found, as determined by one way-Anova.

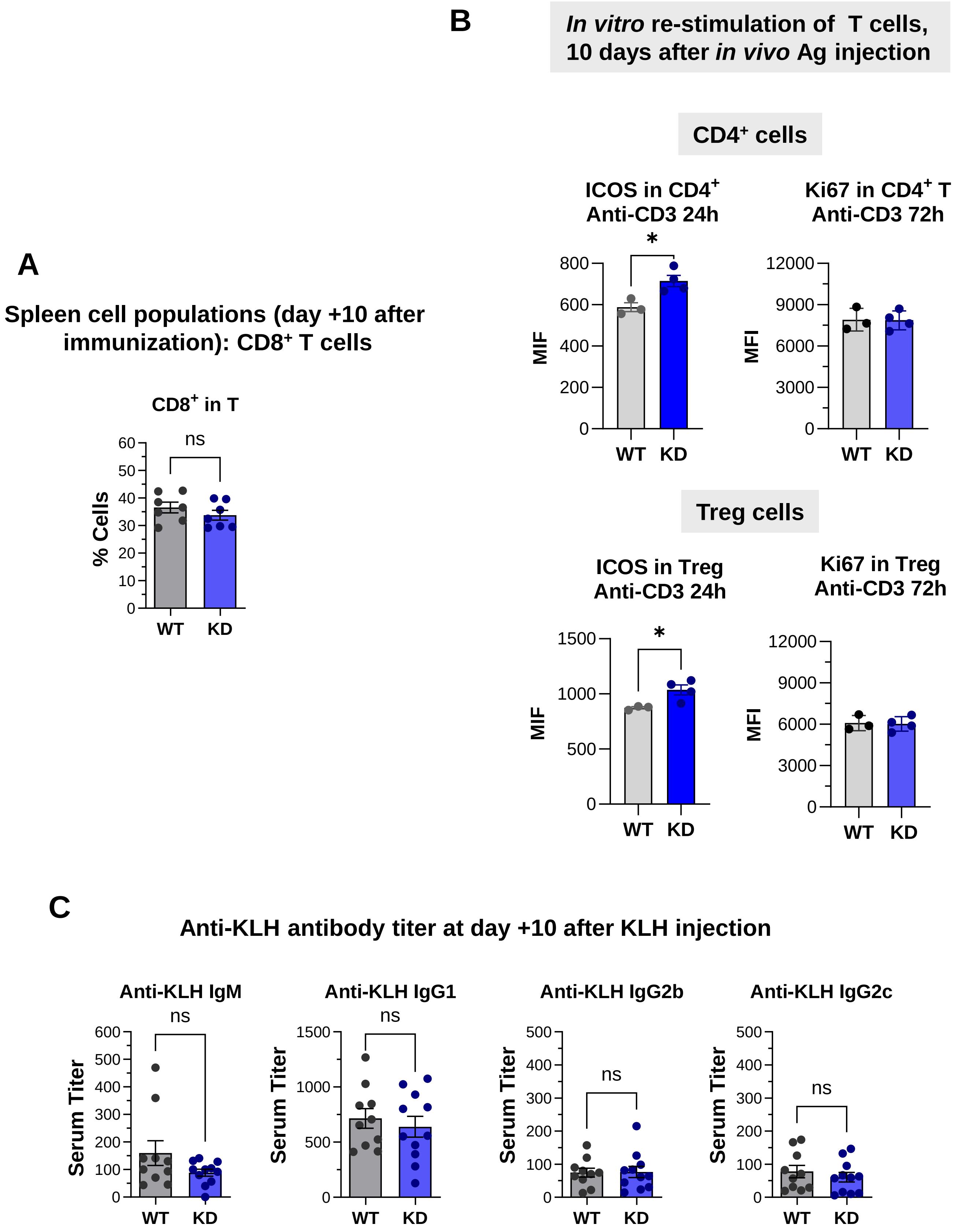

Figure S8. T cell features and anti-KLH specific antibodies in the primary response against KLH (10 days after i.p. immunization with KLH in Alum). (A) Percentage of CD8$^+$ T lymphocytes in the spleen cells of WT (grey) and KD (blue). (**B**) Functional (ICOS, left panels) and proliferative (KI67, right panels) markers in CD4$^+$ spleen T cells (top panels) or CD4$^+$CD25$^+$Foxp3$^+$ Treg spleen cells (bottom panels) from WT (grey) and KD (blue) mice after culture for 72h in the presence of anti-CD3. MFI, Mean of fluorescence intensity. (**C**) Titer of KLH-specific antibodies of the IgM, IgG1, IgG2b and IgG2c classes and subclasses in WT (grey) and KD (blue) mice. Data from individual WT (grey dots) or p110αKD-T mice (KD, blue dots) and the mean ± SEM for each group are also shown. Significant differences between WT and KD mice determined by the Student´s t test are shown (* $p < 0.05$, ** $p < 0.01$).

Strategy to obtain conditional kinase-dead mouse PI3K p110α

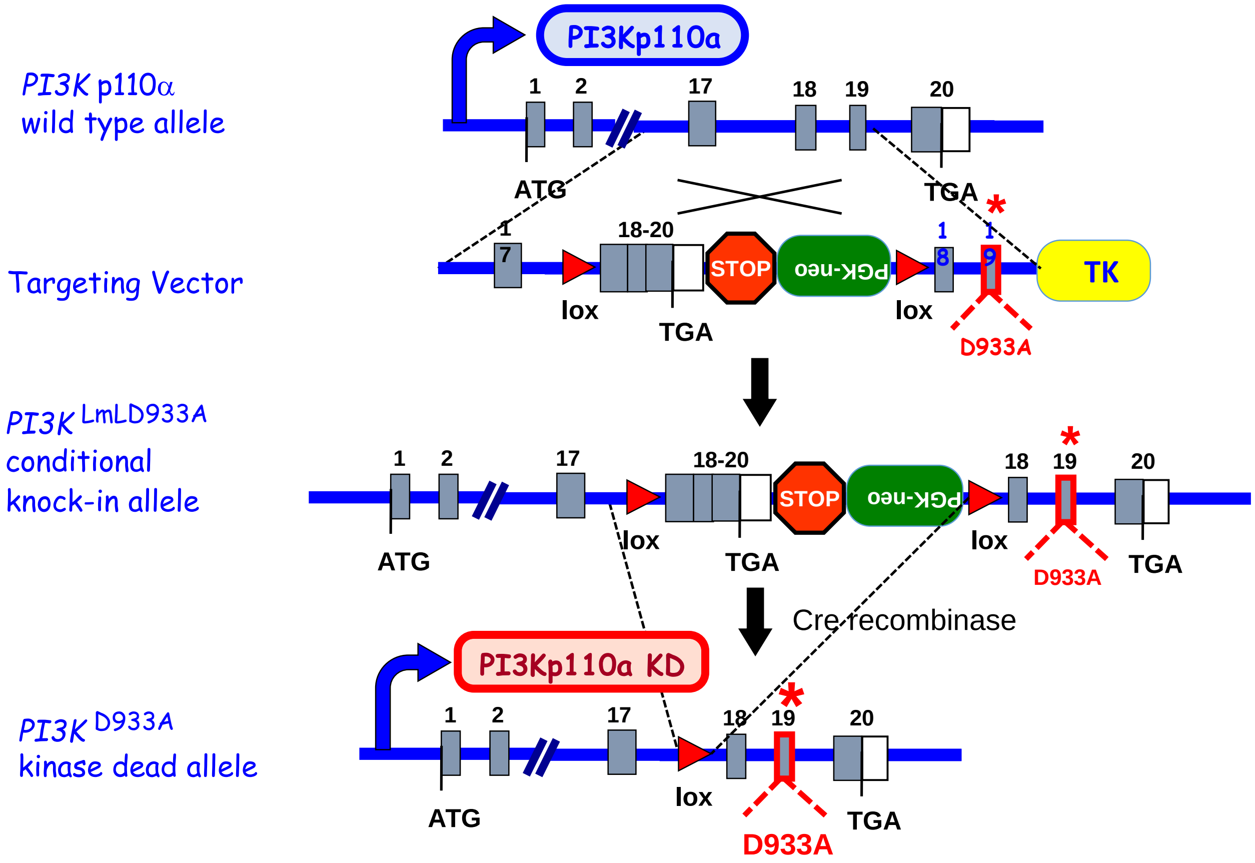

Figure S9.- Genetic structure of transgenic line Pik3ca_LML for conditional kinase dead mutation in the catalytic domain (p110α cKD). (LML, Lox-Minigene-Lox).
See details in Materials and Methods 4.1.

Table S1. *Characteristics of the antibodies and other reagents used in this study.*

Specificity (mouse)	Label	Clone	Isotype	Species	Brand
**AKT1/PKBα**	-	Polyclonal, Ref. 07-416		Rabbit	UpState
**AKT1/PKBα- Phospho Ser473**	-	D9E	IgG	Rabbit	Cell Signalling Technology
**CD3**	APC/Cy7	2C11	IgG	Hamster	BioLegend
**CD3**	-	Y-CD3-1	IgG	Rat	In house, Ref. [60]
**CD4**	PE/Cy7	RM4.5	IgG2a	Rat	eBioscience
**CD4**	APC	RM4-5	IgG2a	Rat	eBioscience
**CD5**	BV711	53-7.3	IgG2a	Rat	BioLegend
**CD8**	BV510	53-6.7	IgG2a	Rat	BioLegend
**CD11b**	APC/Cy7	M1/70	IgG2b	Rat	BioLegend
**CD11c**	PECy7	N418	IgG	Hamster	BioLegend
**CD19**	BV421	6D5	IgG2a	Rat	BioLegend
**CD19**	PECy7	eBio1D3	IgG2a	Rat	eBiosciencie
**CD24**	PE	M1/69	IgG2b	Rat	eBioscience
**CD25**	PE	PC61.5	IgG1	Rat	eBioscience
**CD25**	APC	PC61	IgG1	Rat	BioLegend
**CD28**	-	37.51	IgG	Hamster	eBioscience
**CD44**	FITC	IM7	IgG2b	Rat	BioLegend
**CD62-L**	APC	MEL14	IgG2a	Rat	BioLegend
**CD69**	PE	H1-2F3	IgG	Hamster	BioLegend
**CD69**	BV785	FN50	IgG1	Mouse	BioLegend
**CXCR5**	FITC	SPRCL5	IgG2a	Rat	eBioscience
**ICOS (CD278)**	BV421	C398.4A	IgG	Hamster	BioLegend
**FOXP3**	PE	3G3	IgG1	Mouse	eBioscience
**Ig Isotypes (Mouse IgG1, IgG2b, IgG2c, IgM)**	HRP	Polyclonal, Cat# 1071-05; 1091-05; 1078-05; 1021-05	-	Goat	Southern Biotech.
**IL-4**	-	11B11	IgG1	Rat	eBioscience
**IFNγ**	-	XMG1.2	IgG1	Rat	eBioscience
**Ki67**	AF700	SolA15	IgG2a	Rat	eBiosciencie
**MAP Kinase 2 (Erk2)**	-	Polyclonal, Ref. 06-333		Rabbit	UpState
**MAP Kinase Erk 1/2- Phospho p44/42 (Thr202/Tyr204)**	-	Polyclonal, Ref. 9101		Rabbit	Cell Signalling Technology
**MAP Kinase P38 α/β**	-	Polyclonal, Ref. H-147		Rabbit	Santa Cruz Biotechnology
**MAP Kinase- Phospho P38 α/β (Thr180/Tyr182)**	-	Polyclonal, Ref. 9211		Rabbit	Cell Signalling Technology
**NK1.1**	PECy7	PK136	IgG2a	Rat	eBioscience
**PD-1**	APC	J43	IgG	Hamster	eBioscience
**TCRγδ**	PECy7	eBioGL3	IgG	Hamster	eBioscience
**PNA (Peanut agglutinin)**	FITC	Ref. FL-1071	-	-	Vector
**Rabbit IgG**	HRP	Polyclonal, Ref. A0545	-	Goat	Sigma-Aldrich
**TCRγδ**	PECy7	eBioGL3	IgG	Hamster	eBioscience
**Vav (mouse oncoVav$_{738-845}$-GST)**	-	Polyclonal, affinity purified	-	Rabbit	In house
